# Impaired binding affinity of YTHDC1 with METTL3/METTL14 results in R-loop accumulation in myelodysplastic neoplasms with DDX41 mutation

**DOI:** 10.1038/s41375-024-02228-4

**Published:** 2024-03-21

**Authors:** Won Chan Hwang, Kibeom Park, Silvia Park, Na Young Cheon, Ja Yil Lee, Taejoo Hwang, Semin Lee, Jong-Mi Lee, Min Kyung Ju, Joo Rak Lee, Yong-Rim Kwon, Woo-Lam Jo, Myungshin Kim, Yoo-Jin Kim, Hongtae Kim

**Affiliations:** 1 Department of Biological Sciences, Ulsan National Institute of Science and Technology, Ulsan, Korea; 2 Department of Hematology, Seoul St. Mary’s Hematology Hospital, College of Medicine, The Catholic University of Korea, Seoul, Korea; 3 Leukemia Research Institute, College of Medicine, The Catholic University of Korea, Seoul, Korea; 4 Department of Biomedical Engineering, College of Information and Biotechnology, Ulsan National Institute of Science and Technology, Ulsan, Korea; 5 Catholic Genetic Laboratory Center, Seoul St. Mary’s Hospital, College of Medicine, The Catholic University of Korea, Seoul, Korea; 6 Department of Laboratory Medicine, College of Medicine, The Catholic University of Korea, Seoul, Korea; 7 Department of Orthopaedic Surgery, College of Medicine, The Catholic University of Korea, Seoul, Korea

**Keywords:** Myelodysplastic syndrome, Cell signalling, Cancer genomics

## Abstract

DEAD box helicase 41 (DDX41) mutations are the most prevalent predisposition to familial myelodysplastic syndrome (MDS). However, the precise roles of these variants in the pathogenesis of MDS have yet to be elucidated. Here, we discovered a novel mechanism by which DDX41 contributes to R-loop-induced DNA damage responses (DDR) in cooperation with the m6A-METTL complex (MAC) and YTHDC1 using *DDX41* knockout (KO) and *DDX41* knock-in (KI, R525H, Y259C) cell lines as well as primary samples from MDS patients. Compared to wild type (WT), *DDX41* KO and KI led to increased levels of m6A RNA methylated R-loop. Interestingly, we found that DDX41 regulates m6A/R-loop levels by interacting with MAC components. Further, DDX41 promoted the recruitment of YTHDC1 to R-loops by promoting the binding between METTL3 and YTHDC1, which was dysregulated in *DDX41*-deficient cells, contributing to genomic instability. Collectively, we demonstrated that DDX41 plays a key role in the physiological control of R-loops in cooperation with MAC and YTHDC1. These findings provide novel insights into how defects in DDX41 influence MDS pathogenesis and suggest potential therapeutic targets for the treatment of MDS.

## Introduction

MDS is clonal myeloid neoplasms and predominantly sporadic disease affecting the elderly due to the acquisition of age-related somatic mutations [1–4]. Inherited forms of MDS are increasingly recognized following the advent of genetic screening [5, 6], with *DDX41* the most commonly mutated gene. Since the first report of *DDX41* mutations in myeloid neoplasms [7], both germline and acquired somatic *DDX41* mutations have been identified to define a significant disease entity characterized by late-onset and unique clinical features [8, 9]. The role of *DDX41* in the pathogenesis of myeloid neoplasms has yet to be fully elucidated even though several studies were reported [10–12]. As DDX41 has been shown to interact with core splicing factors (SF) [7, 13] and R-loop-induced genomic instability has been observed in cases of MDS having SF mutations [14–17], there is a need for in-depth studies about the relation of R-loops in association with *DDX41* mutations in MDS.

R-loops are three-stranded nucleic acid structures comprising a DNA-RNA hybrid and a displaced single-stranded DNA [18]. R-loops may interfere with DNA replication, repair, and transcription, thereby compromising genome integrity and function [19]. Recent studies have demonstrated that N^6^-methyladenosine (m6A) modification of RNA moieties regulates the formation and genomic instability of DNA-RNA hybrids [20, 21], and a study reported an association between DDX41 and R-loops [10]. The m6A-methyltransferase complex (MTC) is composed of METTL3 and METTL14 methyltransferases as a catalytic core with several other proteins necessary for catalytic reaction [22, 23]. m6A-modified RNA can be recognized by m6A reader proteins, including the YTH N6-methyladenosine RNA binding proteins (YTHDFs) and YTH domain containing (YTHDCs) families.

This study aimed to determine the role of DDX41 in R-loop physiology and the contribution of DDX41 to m6A methylation of R-loops using *DDX41* knockout (KO) and *DDX41* mutant knock-in (KI) cell lines as well as clinical samples from MDS patients. Furthermore, we evaluated DDR following *DDX41* depletion, which may facilitate the development of novel therapeutic strategies for MDS.

## Materials and methods

### Patient demographics

Genomic data obtained during routine practice in consecutive adult patients (age ≥ 18 years) with MDS according to the WHO 2016 classification between July 2017 and April 2021 at Seoul St. Mary’s Hospital were retrospectively reviewed. The present study was approved by the Institutional Review Board and was conducted according to the Declaration of Helsinki (IRB no. KC22EISI0653 & KC18TESE0700). The details of other experimental methods are described in the supplementary information.

## Results

### Characteristics of patients with *DDX41* mutations

**Fig. 1:**
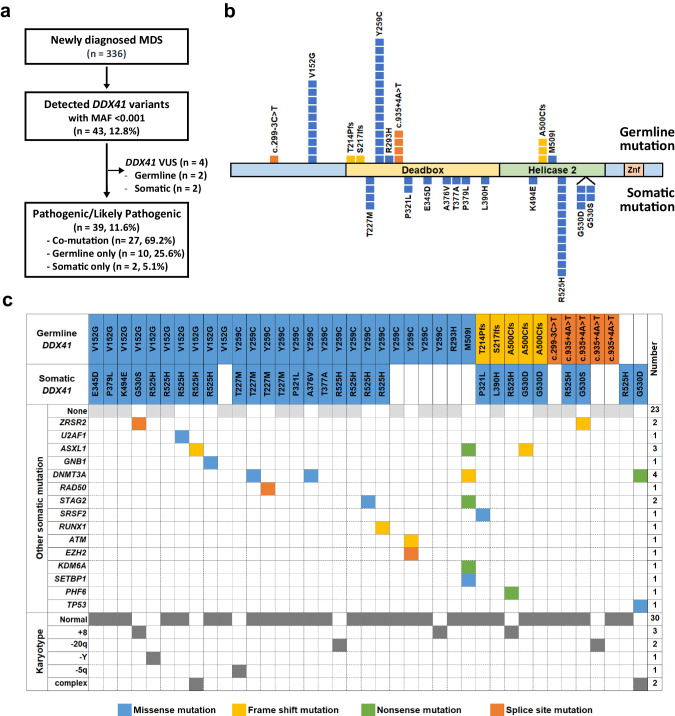
Concurrent mutations and distribution of mutations in Korean MDS patients with *DDX41* mutations. **a** Flow diagram of *DDX41* variants identified in the present study. **b** Graphical representation of the location of germline and somatic *DDX41* mutations identified in the present study against the corresponding DDX41 protein sequence and major functional domains. **c** Germline and somatic *DDX41* mutations, concomitant other recurrent mutations, and karyotypes.

Among a total of 336 patients, 43 cases with *DDX41* variants were detected (Fig. 1a). After the exclusion of four patients with variants of unknown significance (two germline and two somatic variants), a total 66 of pathogenic or likely pathogenic *DDX41* mutations were identified in 39 (11.6%) patients. As shown in Fig. 1b, c, germline and somatic mutations were simultaneously present in 27 patients (69.2%), with the remaining 12 patients having either one germline (*n* = 10, 25.6%) or one somatic mutation (*n* = 2, 5.1%). Of the 37 germline mutations, Y259C was the most common (*n* = 15, 40.5%) followed by V152G (*n* = 10, 27.0%), c.935+4 A > T (*n* = 4, 10.8%), and A500Cfs*9 (*n* = 3, 8.1). Two novel germline mutations, R293H and M509I, were identified. Of the 29 somatic mutations, R525H was the most common (*n* = 12, 41.4%) followed by T227M (*n* = 4), G530D (*n* = 3), G530S (*n* = 2), and P321L (*n* = 2). In patients with *DDX41* mutations, co-mutations were observed in 16 patients with a median number of one per patient (range, 1–5). *DNMT3A* (*n* = 4, 25.0%) and *ASXL1* (*n* = 3, 18.8%) mutations were relatively common. Among the 39 patients, *DDX41* mutations were significantly associated with older age, male sex, normal karyotype, decreased numbers of white blood cells and absolute neutrophils, and higher BM blast percentage (Supplementary Table S1), similar to previous reports [9]. The unique findings of different allele types of *DDX41* mutations with high frequency among Korean MDS patients were also observed [24]. This prompted us to investigate the molecular mechanisms underlying the effects of *DDX41* mutations, frequently observed germline and somatic variants (Y259C and R525H), on MDS biology with focusing on DNA damage [25–28] and R-loop accumulation [10, 12].

### R-loop accumulation in DDX41 mutated cells was associated with m6A methylation

**Fig. 2:**
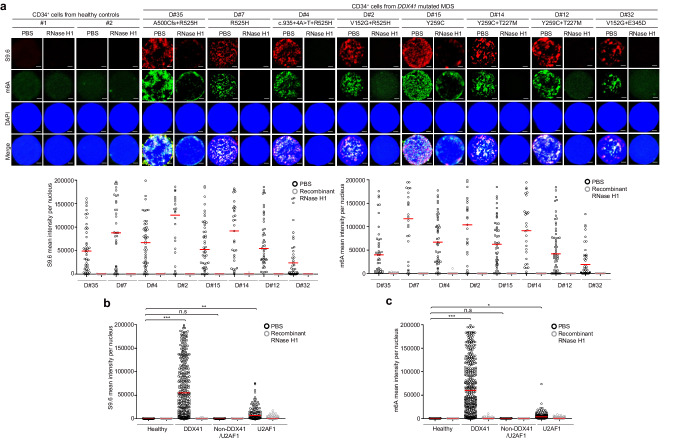
Increased methylated R-loops *DDX41* mutant MDS patients’ bone marrow samples. **a**–**c** S9.6 and m6A fluorescence intensities in CD34^+^ cells isolated from the BM of healthy controls or MDS patients. CD34^+^ cells were treated with PBS or recombinant RNase H1. After 1 h, m6A and S9.6 intensities were determined by immunofluorescence (**a**). We counted 45 cells from patient D#35, 34 cells from patient D#7, 46 cells from D#4, 23 cells from patient D#2, 43 cells from patient D#15, 46 cells from patient D#14, 31 cells from patient D#12, and 46 cells from patient D#32. Scale bar, 1 μm. **b**, **c** Panels represent healthy cells (*n* = 5; 280 cells), cells with *DDX41* mutations (*n* = 8; 314 cells), cells with other mutations (*n* = 3; 150 cells), cells with no mutations (*n* = 4; 200 cells), and cells with *U2AF1* mutations (*n* = 4; 200 cells). Mean fluorescence intensities per nucleus CD34^+^ cells for S9.6 (**b**) and m6A (**c**). Results are presented as the average of three independent experiments. Error bars indicate standard deviation. *P*-value was calculated based on one-way ANOVA in (**b**) and (**c**) (*P < 0.05, **P < 0.01 and ***P < 0.001. n.s. non-significant).

**Fig. 3:**
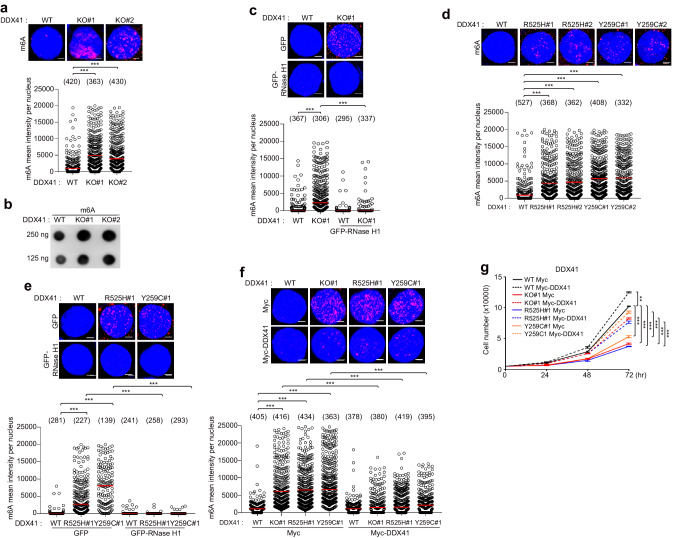
Increased methylated R-loops in *DDX41* mutant cell lines. **a** Quantification of m6A fluorescence intensity was determined by immunofluorescence staining with m6A antibodies in *DDX41* WT and KO K562 cells. **b** m6A dot-blot analysis of *DDX41* WT and KO K562 cells. **c** m6A intensity was reduced in *DDX41* KO K562 cells over-expressing RNase H1. *DDX41* WT and KI K562 cells were transfected with GFP or GFP-RNase H1 expression plasmids. After 48 h, m6A intensity was determined by immunofluorescence. Transfected cells were fixed and stained with m6A antibody. **d** Quantification of m6A fluorescence intensity was determined by immunofluorescence staining with m6A antibodies in *DDX41* WT and KI K562 cells. **e** m6A intensity was reduced in *DDX41* KI K562 cells over-expressing RNase H1. *DDX41* WT and KI K562 cells were transfected with GFP or GFP-RNase H1 expression plasmids. After 48 h, m6A intensity was determined by immunofluorescence. Transfected cells were fixed and stained with m6A antibody. **f**, **g** *DDX41* WT, KO, and KI K562 cells were transfected with Mock or Myc-DDX41 expression plasmids. After 72 h, m6A (**f**) intensity was determined by immunofluorescence. Transfected cells were fixed and stained with m6A antibodies. The numbers above each sample indicate the n value, which is the number of nuclei analyzed. **g** Viability of transfected cells. 5000 cells were plated, and the number of viable cells was counted at indicated time points. Results are presented as the average of three independent experiments. Error bars indicate standard deviation. *P*-value was calculated based on ordinary one-way ANOVA in (**a**, **c**–**f**), two-way ANOVA in (**g**) (**P < 0.01, ***P < 0.001)^.^ Scale bar, 5 μm.

DDX41 belongs to RNA helicase family. Increasing evidence has shown that RNA-related genomic instability is caused mostly by R-loops. Recent studies have highlighted the role of m6A modifications on RNA in governing the formation and genomic instability of R-loops [20, 29]. Therefore, we attempted to analyze the m6A methylation of R-loops using *DDX41* KO, *R525H* or *Y259C DDX41* KI cell lines, and clinical samples from MDS patients. First, we assessed both R-loop accumulation and m6A levels within MDS patients including *DDX41 R525H* or *Y259C* mutation. R-loop and m6A signals were measured in CD34^+^ cells isolated from 19 MDS patients and five healthy controls (Supplementary Table S2). In addition to MDS patients with *DDX41* mutations (*n* = 8), MDS patients with SF mutations (*n* = 4, *U2AF1* mutations in all) and non-*DDX41*/SF mutations (*n* = 7) were also included based on previous studies of R-loop in MDS patients [16, 17]. As shown in Fig. 2a–c and Supplementary Fig. S1, R-loop accumulation and m6A levels were significantly elevated in *DDX41*-mutated CD34^+^ cells compared to healthy CD34^+^ cells. Higher R-loop and m6A levels were observed in CD34^+^ cells from patients with *U2AF1* mutations but not from patients with non-*DDX41*/SF mutations. Since RNase H1 can eliminate DNA-RNA hybrid from R-loops [30], we investigated its potential role in reducing m6A levels within R-loops in CD34^+^ cells. Indeed, R-loops and m6A modifications in CD34^+^ cells from MDS patients were suppressed by treatment with recombinant RNase H1 confirming R-loop formation (Fig. 2a–c). We next analyzed methylated R-loop accumulation using K562 cell lines having *DDX41* KO, *DDX41-R525H*, or *DDX41-Y259C*, which were generated by CRISPR/Cas9 technology (Supplementary Fig. S2a–2c). In our investigation, we established that *DDX41-Y259C* KI clones displayed homozygous mutations, while the *DDX41-R525H* KI clones carried heterozygous mutations. In contrast to WT K562 cells, both *DDX41* KO and KI cell lines exhibited decreased proliferation (Supplementary Fig. S2d, 2e). Confirmatory evidence of escalated DNA damage emerged through the quantification of γH2AX levels in K562 cell lines having *DDX41* KO, *DDX41-R525H* KI, *DDX41-Y259C* KI, and the two mutants overexpressed (Supplementary Fig. S3a–3f). Consistently, we observed a significant increase in m6A levels in *DDX41* KO and KI cells (Fig. 3a–e). m6A modifications were also reduced upon transfection with GFP-RNase H1 in *DDX41* KO and KI cells (Fig. 3c, e), which was accompanied by decreasing γH2AX and R-loop signals (Supplementary Fig. S3g–3i). Subsequent restoration experiments by transfecting Myc-DDX41 into the cells demonstrated reduced m6A, S9.6, and γH2AX levels, as well as restored cell proliferation (Fig. 3f, g, and Supplementary Fig. S3j, 3k). Additionally, we confirmed that m6A modifications co-localized with R-loops in *DDX41* KO and KI K562 cells (Supplementary Fig. S3l, 3m). Reduced expression of DDX41 Y259C mutant was observed in K562, HEK293T, HeLa and patient samples (Supplementary Fig. S3d, S3n–3p). One potential hypothesis is that the Y259C mutation may destabilize the DDX41 structure, leading to increased susceptibility to degradation via proteolytic pathways. Alternatively, the mutation may increase the post-translational modifications essential for DDX41 instability, like ubiquitination. Of note, these hypotheses are speculative at this stage and would require further experiments to elucidate the precise mechanisms underlying the reduction of Y259C-mutated DDX41 protein expression. We also observed a decrease in cell proliferation and accumulation of S9.6 and m6A levels in the MDS-derived cell line SKM-1 upon DDX41 depletion (Supplementary Fig. S4a–4c).

### Direct involvement of DDX41 in DNA damage response by interacting with m6A modified R-loops

**Fig. 4:**
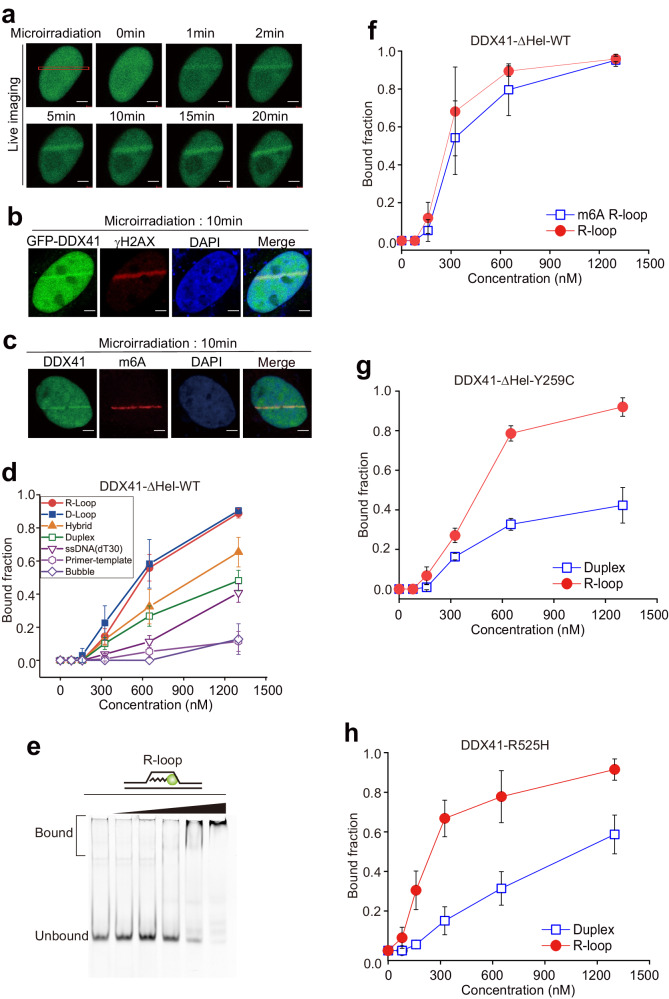
DDX41 translocates to DNA damage sites by binding to R-loops independently of m6A methylation. **a** The translocation of DDX41 to DNA damage sites. HeLa cells expressing GFP-DDX41 were subjected to laser microirradiation. Laser stripes were examined at the indicated time points. Scale bar, 5 μm. **b** HeLa cells expressing GFP-DDX41 were subjected to laser microirradiation. After 10 min, cells were fixed and stained with anti-GFP and anti-γH2AX antibodies. 4,6-diamidino-2-phenylindole (DAPI) was used to stain nuclei. Scale bar, 5 μm. **c** HeLa cells expressing GFP-DDX41 were subjected to laser microirradiation. After 10 min, cells were fixed and stained with anti-GFP and anti-m6A antibodies. 4,6-diamidino-2-phenylindole (DAPI) was used to stain nuclei. Scale bar, 5 μm. **d** Quantification of DDX41-ΔHel-WT EMSA with various DNA substrates. DDX41-ΔHel-WT (0, 80, 160, 325, 650, and 1300 nM) was titrated to 10 nM of DNA or DNA-RNA hybrid substrates. The bound fraction was calculated as the intensity of the bound band divided by the total intensity of all bands. **e** EMSA gel image for DDX41-ΔHel-WT binding to R-loop substrates. DDX41-ΔHel-WT (0, 80, 160, 325, 650, and 1300 nM) was titrated to 10 nM of R-loop substrates. **f** Quantification of DDX41-ΔHel-WT EMSA results with m6A R-loops and unmodified R-loop substrates. DDX41-ΔHel-WT (0, 80, 160, 325, 650, and 1300 nM) was titrated to 10 nM of DNA or DNA-RNA hybrid substrates. **g** Quantification of DDX41-ΔHel-Y259C EMSA results with duplex and R-loop substrates. DDX41-ΔHel-Y259C (0, 80, 160, 325, 650, and 1300 nM) was titrated to 10 nM of DNA or R-loop hybrid substrates. **h** Quantification of DDX41-R525H EMSA results with duplex and R-loop substrates. DDX41-R525H (0, 80, 160, 325, 650, and 1300 nM) was titrated to 10 nM of DNA or R-loop hybrid substrates. The bound fraction was calculated as the intensity of the bound band divided by the total intensity of all bands. Error bars represent the standard deviation of three independent experiments.

As proteins containing a DEAD box and helicase domain are known to migrate to DNA damage sites [31], we examined the DDX41 translocation to DNA damage sites using a laser micro-irradiation assay. The accumulation of GFP-DDX41 at DNA lesions marked with γH2AX peaked at approximately 5 min and persisted at later time points (Fig. 4a, b). We also detected the colocalization of DDX41 and m6A (Fig. 4c), indicating that DDX41 may translocate to m6A-associated DNA damage sites. Next, we attempted to identify the domain responsible for DDX41 migration. Cells with GFP-DDX41 WT, D1, D3, and D4 deletions were recruited to DNA lesions, whereas deletion of the DEAD box (GFP-DDX41 D2) was not (Supplementary Fig. S5a–5d). We also observed accumulation of DDX41-DEAD to DNA damage sites (Supplementary Fig. S5e–5g). In the laser micro-irradiation experiments with DDX41 having a specific point mutation at Y259C or R525H, the mutant DDX41 gathered at DNA damage sites (Supplementary Fig. S5h and 5i), indicating that DDX41 mutations do not affect the DDX41 translocation to DNA damage sites. To biochemically investigate the binding between DDX41 and R-loops at sites of DNA damage, we purified DDX41_ΔHel_WT (DDX41 D3; Supplementary Fig. S6a). Using the purified DDX41 mutant, we performed EMSA with different types of DNA substrates (Fig. 4d, e, Supplementary Fig. S6b–6g). We found that DDX41_ΔHel_WT preferentially binds to R-loops and D-loops with minimal binding to bubble structures, indicating that DDX41 recognizes triple-stranded structures. m6A had no effect on binding affinity, suggesting that the recognition of R-loops by DDX41 is independent of m6A (Fig. 4f and Supplementary Fig. S6h, 6i). As with WT DDX41, mutant DDX41 (Y259C and R525H) preferentially bound to R-loops (Fig. 4g, h and Supplementary Fig. S6j–6o).

### Novel interaction of DDX41 with YTHDC1, METTL3, and METTL14

**Fig. 5:**
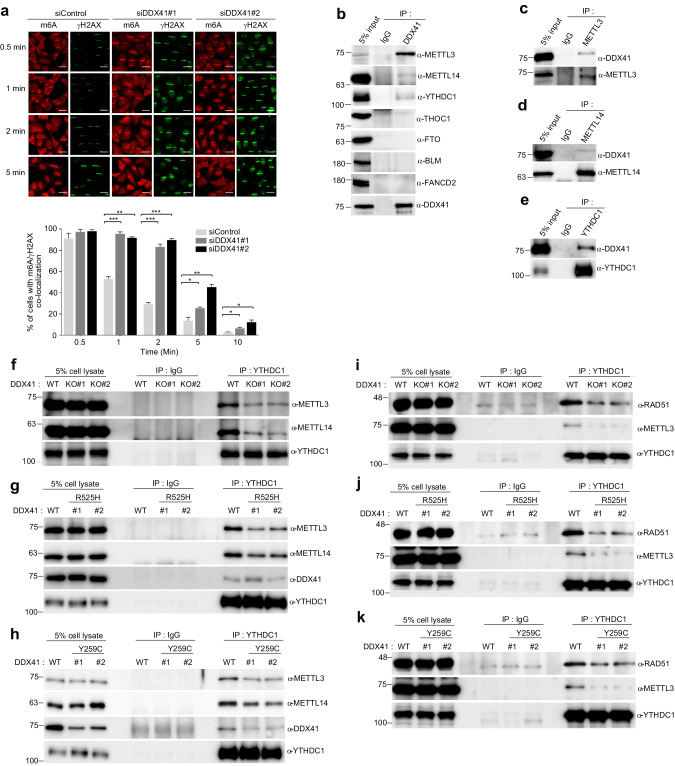
DDX41 binds to YTHDC1, METTL3, and METTL14. **a** m6A fluorescence intensity at DNA damage sites was reduced in *DDX41* knockdown HeLa cells. HeLa cells transfected with control or *DDX41* siRNA were subjected to laser microirradiation. After the indicated durations, cells were fixed and stained with anti-m6A and anti-γH2AX antibodies. The remaining efficacy of m6A in DNA damage site represented the percentage of co-localizing with γH2AX-positive damage sites in cells. Data are presented as the mean ± SEM of three independent experiments. *P*-value was calculated based on one-way ANOVA in (**a**) (*P < 0.05, **P < 0.01, ***P < 0.001). Scale bar, 20 μm. **b**–**e** Interactions between DDX41 and R-loop factors (THOC1, BLM, FANCD2), and MACOM (METTL3, METTL14, YTHDC1, FTO). Immunoprecipitation was performed using control IgG or indicated antibodies and subjected to Western blotting using indicated antibodies. **f**–**k** The interactions between YTHDC1/METTL3 or /METTL14 (**f**–**h**), and YTHDC1/RAD51 or /METTL3 (**i**–**k**) in *DDX41* KO or KI K562 cells. Immunoprecipitation was performed using rabbit IgG or indicated antibodies and subjected to Western blotting using indicated antibodies.

In an effort to assess the mechanism by which DDX41 regulates methylated R-loops, we investigated the kinetics of m6A when DDX41 was knocked down. We observed prolonged m6A persistence at laser stripes in siDDX41 cells compared to the control group (Fig. 5a). Subsequently, we examined the interaction between DDX41 and R-loop factors (THOC1, BLM, FANCD2), METTL3, METTL14, YTHDC1, and FTO. DDX41 was found to bind to METTL3, METTL14, and YTHDC1 by co-immunoprecipitation (Fig. 5b–e). To identify the regions of DDX41 that facilitate this binding, we generated Myc-DDX41 deletion mutants (DDX41-A1 to -A4, -N, and -C) (Supplementary Fig. S7a). Co-immunoprecipitation and immunoblotting analyses demonstrated that the N-terminus of DDX41 interacts with METTL3, METTL14, and YTHDC1 (Supplementary Fig. S7a–7d). In the reciprocal experiment, we found that the methyltransferase domains of METTL3 and METTL14, and YTH domain of YTHDC1 (Supplementary Fig. S7e–7p) interacted with DDX41. These results demonstrated that METTL3, METTL14, and YTHDC1 are interaction partners of DDX41 that have not been recognized. This is a new finding in this study.

### Function of DDX41 in mediating the binding between METTL3/METTL14 and YTHDC1

Subsequently, we focused on deciphering the mechanisms through which DDX41, METTL3, METTL14, and YTHDC1 exert regulatory control over m6A signaling within the context of R-loop-mediated DNA damage. To this end, we first checked the interactions between the proteins when DDX41 was deficient. The binding affinity between METTL3 and METTL14 (Supplementary Fig. S8a–8c), as well as between METTL3 and DDX41-R525H (Supplementary Fig. S8d), remained unaltered in *DDX41* KO and KI cells compared to *DDX41* WT cells. However, the binding affinity between METTL3 and DDX41-Y259C was diminished compared to DDX41 WT, likely attributable to the reduced expression of *DDX41*-Y259C (Supplementary Fig. S8e). Additionally, the interactions of YTHDC1 with METTL3 or METTL14 displayed decreased binding affinities in *DDX41* KO and KI cells compared to *DDX41* WT cells. Interestingly, the binding affinity between YTHDC1 and DDX41 remained unchanged regardless of the mutational status of DDX41 (Fig. 5f–h), indicating a possible role for DDX41 as a bridge between YTHDC1 and METTL3/METTL14. Furthermore, we observed decreased binding affinities between YTHDC1 and RAD51 in *DDX41* KO and KI cells compared to *DDX41* WT cells (Fig. 5i–k) and had the same result in *DDX41* depleted SKM-1 cells (Supplementary Fig. S9).

### Role of DDX41 in homologous recombination

**Fig. 6:**
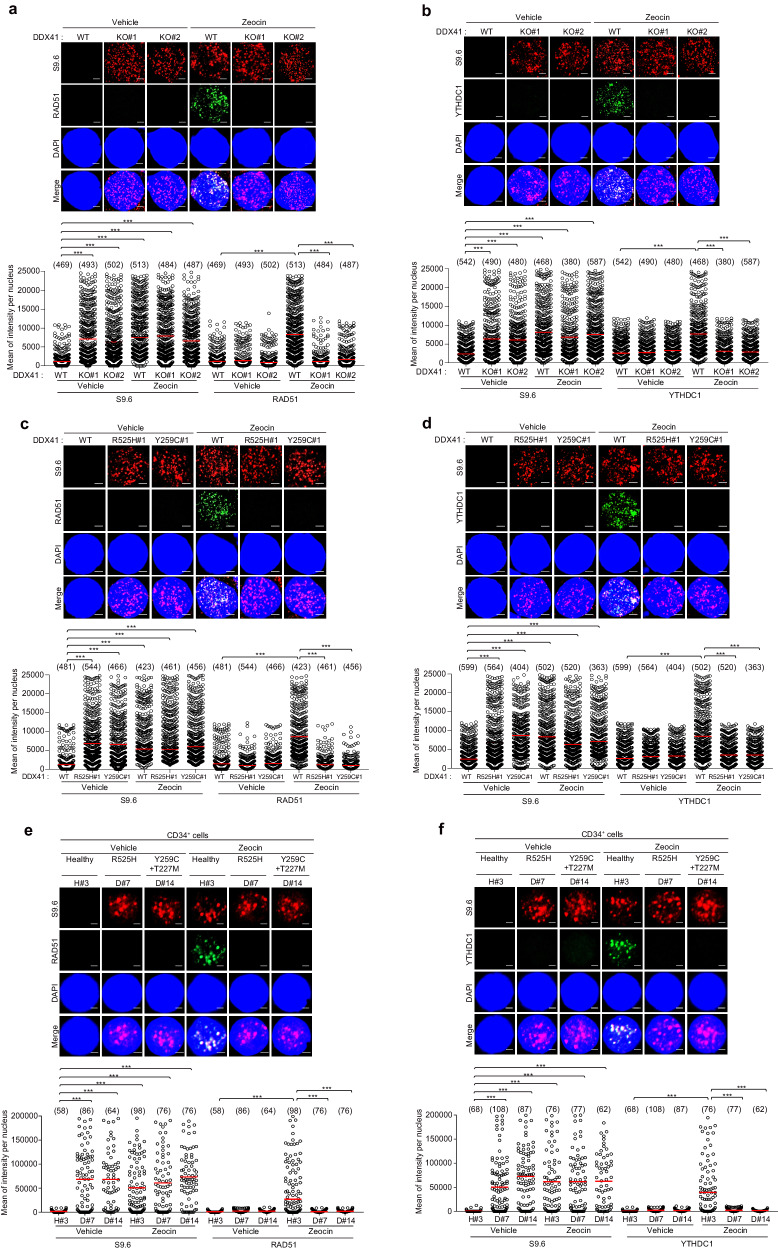
DDX41 directly recruits YTHDC1 to DNA damage sites by mediating the interaction between METTL3 or RAD51 and YTHDC1. **a**, **b** Mean fluorescence intensities of S9.6/RAD51/YTHDC1 were determined by immunofluorescence. *DDX41* WT and KO K562 cells were treated with Vehicle (DMSO) or Zeocin. After 6 h, the cells were with fixed and stained with indicated antibodies. **c**, **d** Mean fluorescence intensities of S9.6/RAD51/YTHDC1 were determined by immunofluorescence. *DDX41* WT and KI K562 cells were treated with Vehicle (DMSO) or Zeocin. After 6 h, the cells were with fixed and stained with indicated antibodies. The numbers above each sample indicate the *n* value, which is the number of nuclei analyzed. **e**, **f** Mean fluorescence intensities of S9.6/RAD51/YTHDC1 were determined by immunofluorescence. CD34^+^ cells isolated from the BM of healthy controls or MDS patients were treated with Vehicle (DMSO) or Zeocin. After 6 h, CD34^+^ cells were fixed and S9.6/RAD51 (**e**) and S9.6/YTHDC1 (**f**) fluorescence intensities were determined by immunofluorescence. We counted indicated cells from healthy controls #3 and patients D#7, and D#14. *P*-value was calculated based on one-way ANOVA in (**a**–**f**) (***P < 0.001). Scale bar, 5 μm (**a**–**d**), 1 μm (**e**, **f**).

R-loops can cause genomic instability by inducing double-strand breaks (DSBs) and replication stress [19]. Reversely, DSBs facilitate the formation of DNA-RNA hybrids [32]. However, the effect of DNA-RNA hybrids on the homologous recombination (HR) process is poorly understood. Further insights came from studying DDX41’s role in HR, a repair mechanism for R-loop-mediated DNA damage [29]. Through a DR-GFP-based HR reporter assay, we observed a 20% decrease in HR efficiency in *DDX41*-depleted cells (Supplementary Fig. S10a). To exclude the off-target effects of *DDX41* siRNAs, we carried out restoration experiments using the siRNA-resistant DDX41 expression plasmid (Supplementary Fig. S10b). Reintroducing the siRNA-resistant Myc-tagged DDX41-WT into cells depleted with endogenous DDX41 successfully rescued the HR capacity (Supplementary Fig. S10c). However, we found that Myc-tagged DDX41-R525H and -Y259C failed to rescue HR. Collectively, our results indicate that DDX41 dysfunction contributes to increased genomic instability in K562 cells and MDS patient samples. Recently, METTL3-m6A-YTHDC1 axis was found to bolster the recruitment of HR repair complex to DNA damage sites in DDX41 WT cells [29]. The YTHDC1 protein, which recognizes m6A modifications, played a protective role for DNA-RNA hybrids at DSBs sites, facilitating the recruitment of RAD51. Our inference suggests that m6A-recognizing YTHDC1 fosters the loading of RAD51, guiding its incorporation into the DNA-RNA hybrid strand. Consequently, the role of DDX41 within the METTL3-m6A-YTHDC1 axis becomes recognized as a linker between METTL3 and YTHDC1. Subsequently, our investigation led to the formation of YTHDC1 and RAD51 foci induced by Zeocin treatment in *DDX41* WT, KO, and KI K562 cells. Intriguingly, while Zeocin treatment prompted the emergence of YTHDC1 and RAD51 foci in *DDX41* WT cells, such formations were absent in *DDX41* KO and KI K562 cells (Fig. 6a–d) and had the same result in *DDX41* depleted SKM-1 cells (Supplementary Fig. S11a). Notably, this outcome persisted even though the accumulation of R-loops was detected in all three types of K562 cells (*DDX41* WT, KO, and KI). Comparable findings were also replicated in *DDX41*-mutant CD34^+^ cells in contrast to healthy CD34^+^ counterparts (Fig. 6e, f and Supplementary Fig. S11b and 11c). Interestingly, we found increased RAD51 and YTHDC1 foci in Zeocin-treated *U2AF1*-mutant CD34^+^ cells, unlike the results obtained from *DDX41*-mutant CD34^+^ cells (Supplementary Fig. S11d and 11e). These results suggest an emerging function of DDX41 in mediating the m6A writer (METTL3 and METTL14) and reader (YTHDC1), which could be disrupted and serve as a primary mechanism for genetic instability in *DDX41*-mutated MDS.

### YTHDC1-METTL3 fusion protein rescues the DNA damage in *DDX41* KO and KI K562 cells

**Fig. 7:**
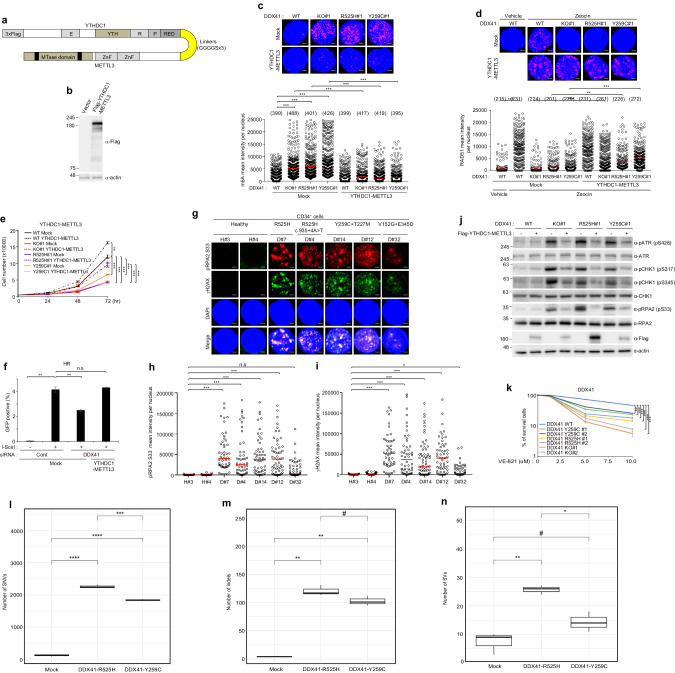
METTL3-YTHDC1 fusion protein rescues the DNA damage in *DDX41* KO and KI K562 cells. **a** Design of YTHDC1-METTL3 fusion proteins and schematic of fusion protein. 3 X Flag tags were conjugated to the N-terminal of protein of YTHDC1. The YTHDC1-METTL3 fusion with a 3 X 8aa linker sequence (GGGGS). **b** The expression validation of Mock or Flag-YTHDC1-METTL3fusion protein by western blotting. **c**, **d** *DDX41* WT, KO, and KI K562 cells were transfected with Mock or YTHDC1-METTL3 expression plasmids. After 72 h, m6A (**c**) and RAD51 (**d**). The numbers above each sample indicate the *n* value, which is the number of nuclei analyzed. **e** Viability of transfected cells. The transfected 5000 cells were plated, and the number of viable cells was counted at indicated time points. Results are presented as the average of three independent experiments. Error bars indicate standard deviation. **f** Measurement of homologous recombination capacity in DR-GFP reporter U2OS cells. U2OS cells harboring the DR-GFP reporter were treated with the indicated siRNAs, followed by transfection with the indicated expression plasmids. Two days later, GFP expression was accessed by flow cytometry. The results represent the average of three independent experiments. The error bars indicate the standard deviation. **g**–**i** CD34^+^ cells were fixed and pRPA2 S33 and γH2AX fluorescence intensities were determined by immunofluorescence. We counted 50 cells from healthy controls #1 and #2 and patients D#7, D#4, D#14, D#12, and D#32. **j** Mock or YTHDC1-METTL3 expression plasmids transfected *DDX41* WT, KO and KI K562 cell lysates were immunoblotted with indicated antibodies. **k** Viability of WT, *DDX41* KO, and *DDX41* KI K562 cells following treatment with an ATR inhibitor (VE-821). 5000 cells were plated and treated with increasing concentrations of VE-821 (0, 1.25, 2.5, 5, and 10 μM). The number of cells was counted culture with VE-821 for four days. Data are presented as the mean ± SEM of three independent experiments. Scale bar, 5 μm (**c**, **d**) 1 μm (**g**). **l** The statistical significance of the differences in single nucleotide variant numbers among the K562 cells is shown in a box plot. **m** The statistical significance of the differences in short insertion and deletion numbers among the K562 cells is shown in a box plot. **n** The statistical significance of the differences in structural variation numbers among the K562 cells is shown in a box plot. *P*-value was calculated based on one-way ANOVA in (**c**–**f**, **h**, **i**), two-way ANOVA in (**k**), and a Student’s *t*-test in (**l**–**n**) (one-way ANOVA and two-way ANOVA; *P < 0.05, **P < 0.01 and ***P < 0.001. n.s. non-significant, Student’s *t*-test; ^#^*P* < 0.1, *P < 0.05, **P < 0.01, ***P < 0.001, ****P < 0.001).

As previously suggested, the critical role of DDX41 in facilitating the connection between METTL3 and YTHDC1 was disrupted by the gene mutation. To test the significance of these disruptions in controlling m6A-methylated R-loops, restoration experiments were carried out. For this purpose, chimeric fusions, encompassing the genes encoding METTL3 with YTHDC1, were generated (Fig. 7a, b), and these constructs were transfected into *DDX41* KO and KI K562 cells, and SKM-1 cells. Flag-YTHDC1-METTL3 transfected cells showed the reduction of DNA damage, cell proliferation and HR (Fig. 7c–f and Supplementary Fig. S12a–12e). As R-loop formation is critical for activating ATR/CHK1 signaling to stabilize and protect stalled replication forks under replication stress [14, 33], we evaluated the effect of *DDX41* mutation on ATR activation. We observed increased levels of phosphorylated ATR (S428), CHK1 (S317 and S345), and RPA2 (S33), which are well-known markers of R-loop-mediated replication stress, in *DDX41* KO and KI cells (Supplementary Fig. S13a, 13b). Over-expression of RNase H1 inhibited the activation of the ATR pathway, confirming that replication stress in *DDX41* KO or KI cells is due to the accumulation of R-loops (Supplementary Fig. S13c, 13d). We also detected levels of phosphoRPA2 (S33) were significantly higher in CD34^+^ cells with *DDX41* mutations compared to healthy CD34^+^ cells (Fig. 7g–i). Further restoration experiments through the transfection of Myc-DDX41 or Flag-YTHDC1-METTL3 showcased reduced phosphorylation levels of ATR (S428), CHK1 (S317 and S345), and RPA2 (S33) (Fig. 7j and Supplementary Fig. S13e, 13f). Next, our observations revealed that *DDX41* KO, KI, and *DDX41* depleted SKM-1 cells showed increased sensitivity to ATR inhibitor (VE-821, Fig. 7k, Supplementary Fig. S13g). However, the introduction of Flag-YTHDC1-METTL3 into these cells reversed this increased sensitivity (Supplementary Fig. S13h). Furthermore, healthy CD34^+^ cells transfected with *DDX41*-R525H or -Y259C displayed resolved S9.6 and phospo-RPA2 signals after overexpression of the YTHDC1-METTL3 fusion protein (Supplementary Fig. S14a–14e). Our results provide experimental evidence that YTHDC1-METTL3 participates in the clearance of R-loops, though the underlying mechanism remains unclear. To explore this further, we investigated that YTHDC1-METTL3 complex recruits RNase H1 to degrade R-loops. Our results revealed that the translocation of GFP-RNase H1 D210N to DNA damage sites was reduced in *DDX41*-depleted HeLa cells, and this effect was rescued by the Flag-YTHDC1-METTL3 protein (Supplementary Fig. S15).

### Whole-genome sequencing of genomic instability in the *DDX41* knock-in cell lines

To investigate genomic instability in the *DDX41* KI K562 cells, we conducted whole-genome sequencing. Single nucleotide variants (SNVs) were significantly increased in the two *DDX41* mutant groups than in the control samples (Fig. 7l). Notably, the largest number of SNVs was observed in the *DDX41*-R525H group. Furthermore, we also found a higher number of short insertions and deletions (indels) in both *DDX41* mutant groups than in the control samples (Fig. 7m). The indel difference between the two *DDX41* mutant groups was not as significant as the SNVs difference. In contrast to indels and SNVs, structural variations showed a significant increase only in the *DDX41*-R525H group compared to the control samples (Fig. 7n).

## Discussion

DDX41 was previously identified as a mediator of genomic stability [34], and subsequent studies showed that DDX41 dysfunction causes DNA damage by inducing R-loops accumulation [11, 12, 28, 35]. It has remained unclear whether these events manifest similarly in human samples and how mutations in the *DDX41* gene contribute to the accumulation of R-loops. Our study firstly demonstrates that m6A-modified R-loops and DNA damage are elevated in *DDX41*-mutated CD34^+^ cells of MDS patients. Moreover, we unveil a novel mechanism illustrating how DDX41 collaborates with METTL3, METTL14, and YTHDC1 to regulate R-loops, which potentially serve as a foundational factor in the development of MDS through *DDX41* mutations. Furthermore, we observed a significant prevalence of *DDX41* mutations exceeding 10% among Korean patients with MDS, alongside the presence of distinct mutant alleles.

The main focus of our study was to determine the mechanisms underlying the accumulation of R-loops induced by *DDX41* mutations in MDS. We initially assessed the direct involvement of DDX41 in DDR. WT DDX41 was relocated to irradiation-induced damage sites, and mutations had no effect on the translocation capability. An increased level of m6A at R-loops in DDX41 KO and KI cell lines, as well as in MDS samples with *DDX41* mutations, prompted us to explore the binding partners of DDX41 among m6A-associated proteins. The most intriguing findings of this study are the direct engagement of DDX41 with METTL3, METTL14, and YTHDC1 and the new function of DDX41 for mediating the interaction between YTHDC1 and either METTL3 or METTL14. However, we did not detect YTHDC1 or RAD51 localization at R-loop sites in DDX41 KO and KI cells or CD34^+^ cells of *DDX41*-mutated MDS. One potential reason for this observation is the decrease in binding affinity of YTHDC1 with METTL3 or METTL14. Possible explanation we proposed is that YTHDC1 rapidly dissociated from DNA damage sites in the presence of mutant DDX41, leading to reduced YTHDC1 persistence and RAD51 recruitment at R-loop sites. A previous study reported that the METTL3-m6A-YTHDC1 axis promotes the recruitment of BRCA1/RAD51 to DNA damage sites to maintain R-loop stability [29]. The distinctive implication of our study lies in the fact that the successful clearance of R-loops requires the bindings of the m6A writers (METTL3/METTL14) and the m6A reader (YTHDC1) and this process is mediated by DDX41. In contrast to YTHDC1’s established role in the recognition of m6A for the repair process [36], the distinct impact of its direct binding to m6A-writing complexes (METTL3 and METTL14) needs to be revealed.

Our data reveal a new bridging role of DDX41 between YTHDC1 and METTL3, which is unexpected because METTL3-DDX41 and DDX41-YTHDC1 interactions remain intact in R525H mutant K562 cells although METTL3-YTHDC1 interaction is disrupted. This appears to challenge our proposed role of DDX41 in linking the two proteins. One plausible explanation is the potential involvement of DDX41-binding proteins that may be essential for the bridging function. However, the DDX41 binding protein(s) might not interact with DDX41 mutants effectively, thereby impeding DDX41 from the connection between YTHDC1 and METTL3. Consequently, R525H mutation could compromise the bridging function, leading to the disruption of the METTL3-YTHDC1 interaction. Despite the aforementioned hypotheses, we think that further investigation is required to understand the precise mechanism governing the bridging function of DDX41 between YTHDC1 and METTL3.

MDS and acute myeloid leukemia are associated with increased DNA damage and altered DDR [25–27]. The demonstration of genetic mutation as a cause of DNA damage in MDS was first observed in MDS with SF mutations (*SRSF2, U2AF1*, and *SF3B1*) [14–16]. The *DDX41* mutations currently represent the second genetic alteration where R-loop-induced DNA damage has been identified in human MDS samples, supporting the previous findings in cell lines or animal models with *DDX41* mutations [11, 12]. In our study, CD34^+^ cells with *U2AF1* mutations also exhibited an increased level of m6A modifications at R-loops, although this level was lower than that observed in CD34^+^ cells with *DDX41* mutations. Both DDX41 and SF genes are involved in RNA splicing. However, they exhibit significant differences in onset phenotypes and clinical outcomes although their mutations share a commonality in inducing m6A-R-loops-induced DNA damage [37]. In addition to elucidating the pathological mechanisms driven by each genetic mutation [11, 35, 38], revealing the commonalities and distinctions in disease biology between these two groups of genetic mutations will further deepen our comprehension of the pathophysiology of MDS. Understanding the disorder’s molecular mechanisms is closely linked to improving treatment strategies. We found that *DDX41*-mutated cells, like in SF-mutated MDS [39], showed heightened sensitivity to ATR inhibitors. Regarding the role of m6A methylation in leukemogenesis, it has been suggested that targeting m6A regulators may have therapeutic potential and could be considered for the treatment of *DDX41*-mutated MDS [30, 40, 41]. Therefore, there is a compelling need for further exploration into the study of m6A-associated molecules in MDS patients and their potential connections to R-loops.

The current study unveiled a significant frequency of *DDX41* mutations in Korean MDS patients (11.6% in our cohort vs. 0.8–3.9% in other countries), which corroborates earlier observations in Korean patients [24]. While DDX41-R525H was the most common somatic mutation in our study, consistent with other reports, our germline mutations differed by the notable prevalence of Y259C (40.5%) and V152G (24.3%) [5, 8, 24, 42–44]. While observing variations in germline mutation allele types between Korean and non-Korean cohorts, we noted that clinical characteristics typical of DDX41-mutated MDS, such as male predominance, normal karyotype, higher blast percentage, and a favorable response to hypomethylating agents, were also evident in this cohort. In this study, we conducted analyses on representative somatic and germline mutations, namely R525H and Y259C. We did not observe distinctions in the m6A-METTL-YTHDC1 pathway beyond differences in DDX41 protein expression between these two mutations. Another exception is an increased number of SNVs and structural variations in *R525H* KI cells compared to those of *Y259C* KI cells, the clinical significance of which remains to be explored. Regarding the influences of mutated allele types, a recent report has highlighted that truncating variants pose a higher risk of leukemic transformation compared to non-truncating variants among germline mutations [37]. Exploring distinct clinical significance linked to molecular mechanisms of individual variations remains a compelling direction in medical research.

Collectively, the findings of the present study demonstrate that DDX41 plays a key role in the physiological control of R-loops in cooperation with m6A and MTC. Dysregulation of this process in DDX41-deficient cells results in failed recruitment of YTHDC1 and downstream DDR signaling molecules resulting in DNA damage. Due to increased R-loop-mediated replication stress, DDX41-deficient cells have greater ATR-CHK1 activation and enhanced sensitivity to ATR inhibitors. The most crucial function impaired by genetic mutations in *DDX41*, as newly discovered in this study, is its role as a mediator for the interaction between YTHDC1 and either METTL3 or METTL14. The results of the present study provide insights into the molecular mechanisms underlying the pathogenesis of *DDX41* mutated MDS.

### Supplementary information

Supplementary Information

## Data Availability

All of the MDS patient baseline characteristics and genomic data that were obtained during this study are presented in Supplementary Tables 1 and 2. The raw sequencing data are available for download from the Sequence Read Archive (SRA) under accession PRJNA1068287. All other data supporting the findings of this study are available from the corresponding author upon reasonable request.
