# Supplementary material for: Impaired binding affinity of YTHDC1 with METTL3/METTL14 results in R-loop accumulation in myelodysplastic neoplasms with DDX41 mutation
Source: Leukemia. 2024 Mar 21;38(6):1353–64. doi: 10.1038/s41375-024-02228-4 (PMC11147762; doi:10.1038/s41375-024-02228-4)
Supplement: Supplementary file 1 — Supplementary Information [file 41375_2024_2228_MOESM1_ESM.docx]

**Supplementary Information for**

**Impaired binding affinity of YTHDC1 with METTL3/METTL14 results in R-loop accumulation in myelodysplastic neoplasms with DDX41 mutation**

Won Chan Hwang^1,8^, Kibeom Park^1,8^, Silvia Park^2,3,8^, Na Young Cheon^1,8^, Ja Yil Lee^1,8^, Taejoo Hwang^4^, Semin Lee^4^, Jong-Mi Lee^5,6^, Min Kyung Ju^1^, Joo Rak Lee^1^, Yong-Rim Kwon^3^, Woo-Lam Jo^5,7^, Myungshin Kim^5,6,*^, Yoo-Jin Kim^2,3,*^, and Hongtae Kim^1,*^

^*^Corresponding authors. Email: khtcat@unist.ac.kr, yoojink@catholic.ac.kr, microkim@catholic.ac.kr

**This PDF file includes:**

Supplementary Materials and Methods

Supplementary Figures and legends (Supplementary Figure S1 to S15)

Supplementary Tables (Supplementary Tables S1 to S5)

**Supplementary Materials and Methods**

**Genomic data**

Targeted next generation sequencing using bone marrow (BM) samples was performed according to previously described methods (1). *DDX41* variants with a variant allele frequency greater than 0.4 were considered germline variants and were confirmed by analysis of oral epithelial cells. The pathogenicity of detected *DDX41* germline and somatic variants was determined according to the guidelines of the American College of Medical Genetics and Genomics (1) and the Association for Molecular Pathology (2) along with the methods described in previous studies (3-5).

**Isolation of CD34^+^ cells**

Bone marrows (BM) were obtained from MDS patients at the time of diagnosis, and BM or mobilized peripheral blood (PB) were collected from healthy controls with informed consent for research purposes. Frozen cells were thawed in RPMI1640 (Cytiva, Marlborough, MA) containing 20% fetal bovine serum (FBS; Gibco, Franklin Lakes, NJ) at 37°C and treated with DNase 1 (100 U/ml, Thermo Fisher Scientific, Carlsbad, CA) in Dulbecco’s phosphate buffered saline (DPBS; pH 7.0-7.2, Cytiva) containing 0.5 mM MgCl_2_, 1 mM CaCl_2_, and 1% FBS for 60 mins at room temperature (RT). CD34^+^ cells were isolated using human CD34 MicroBead Kits (Miltenyi Biotec, Bergisch Gladbach, Germany), according to the manufacturer’s protocol. The purity of isolated CD34^+^ cells was measured by flow cytometry (CD34-FITC, clone: AC136, Miltenyi Biotec) and was >95% for all tested samples.

**Cell culture**

The SKM-1 cell line, established from a peripheral blood sample at the overt leukemia stage arising in MDS, was purchased from the Japanese Collection of Research Bioresources Cell Bank (Ibaraki, Osaka, Japan). The human chronic myeloid leukemia cell line K562 was purchased from American Type Culture Collection (Manassas, VA). Cell lines were maintained in RPMI1640 (Cytiva) supplemented with 10% FBS (Gibco, Franklin Lakes, NJ) and 1% penicillin/streptomycin (Gibco) in 5% CO_2_ in a 37°C incubator. Cell lines were checked for Mycoplasma contamination using PrepSEQ™ 1-2-3 Mycoplasma Nucleic Acid Extraction Kit (4443789, ThermoFisher Scientific) and tested within every month after cell thawing.

**Plasmids, sgRNAs, siRNAs**

*DDX41* A1, A2, A3, A4, N-term, C-term, and DEAD domain fragment and D1, D2, D3, and D4 deletion mutant expression plasmids were created using GFP-, SFB-, or Myc-tagged mammalian expression vectors. The *DDX41-R525H* and *DDX41-Y259C* point mutants were generated from GFP-tagged *DDX41* using Phusion Site-Directed Mutagenesis Kits (ThermoFisher Scientific, Carlsbad, CA). *METTL3*, *METTL14*, and *YTHDC1* genes were purchased from the Korea Human Gene Bank. SFB-Mettl3, SFB-Mettl14, SFB-D1, SFB-D2, and SFB-D3 deletion mutant expression plasmids were created using a SFB-tagged mammalian expression vector. Myc-YTHDC1, D1, D2, D3, D4, D5, D6, and D7 deletion mutant expression plasmids were created using a Myc-tagged expression vector. GFP-METTL3, and GFP-YTHDC1 expression plasmids were created using GFP-tagged mammalian expression vectors. Generation of YTHDC1-METTL3 fusion protein expression plasmid was created using 3 X Flag tagged mammalian expression vectors. METTL3 conjugated to the C-terminal of protein of YTHDC1. YTHDC1 and METTL3 were linked by a 3 X 8 aa linker sequence (GGGGS). Guide RNA plasmids for human *DDX41* gene were generated by cloning guide sequences into pX330 (plasmid number 42230; Addgene). Target sequences for gene editing were selected using CRISPOR (www.crispor.tefor.net). The sequences of reverse transcription (RT)-PCR primers, siRNAs, sgRNAs, and oligonucleotides for CRISPR/Cas9 knock-in are listed in Supplementary Table S3.

**Transfection of cell lines or CD34^+^ cells**

Transient transfections were performed using the Neon Transfection System (Thermo Fisher Scientific, Carlsbad, CA). The SKM-1, K562 WT, DDX41 deficient, and mutated cell lines (2 × 10^6^ cell/100 μl) were electroporated. A total of 20 μg of transient expression plasmid DNA, guide RNA plasmids, or siRNA were added to cells along with R buffer, and the mixture was electroporated (1450 V for 10 ms in 3 pulses), and incubated in pre-warmed RPMI medium supplemented with 10% fetal bovine serum at 37 °C in 5% CO2 atmosphere for 48~72h. Control and DDX41 siRNA duplexes used in the present can be found in Supplementary Table 3. All experiments were performed at 48~72h after transfection. The thawed or live BM/PB mononuclear cells were cultured for 3 days in RPM11640 supplemented with 10% FBS, 1% penicillin/streptomycin, and 50 ng/mL of myeloid expansion supplements (human interleukin-3, stem cell factor, thrombopoietin and FLT-3 ligand, PeproTech). After 3days, whole cells (5 × 10^6^ cell/100 μl) were electroporated. A total of 20 μg of GFP- or Myc-tagged DDX41 WT or point mutant (R525H, Y259C), and Flag-tagged YTHDC1- METTL3 transient expression plasmid DNAs were added to cells along with T buffer, and the mixture was electroporated (2150 V for 20 ms in 1 pulses), and incubated in pre-warmed RPMI medium supplemented with 10% fetal bovine serum and 50 ng/mL of myeloid expansion supplements at 37 °C in 5% CO2 atmosphere for 72h.

**Generation of *DDX41* knockout (KO) and knock-in (KI) K562 cells**

To generate the *DDX41* KO and *DDX41* KI cell lines, K562 cells were co-transfected with CRISPR/Cas9, double guide RNA targeting *DDX41* or a one-point mutation guide RNA targeting *DDX41,* and donor plasmids using the Neon Transfection system (Thermo Fisher Scientific, Waltham, MA). After 48 h, high mCherry-expressing cells were sorted into 96-well plates using a FACSAria Fusion cell sorter (BD Biosciences, San Jose, CA). *DDX41* KO and KI cell lines were confirmed by targeted sequencing and western blotting for DDX41. Each clone was generated from a single population to avoid clonal heterogeneity.

**Immunofluorescence**

Immunofluorescence (IF) for S9.6, m6A, and co-localization with RAD51, and YTHDC1 were performed as described previously with minor modifications (6). Briefly, each of experiment cells were pelleted in 15 ml tubes at 1200 rpm for 5 min at 25°C. Media was removed until 0.5 ml media remained, and cell pellets were resuspended. Pre-warmed 75 mM KCl solution at 37°C was added to cells in a drop-wise manner while cells were agitated on a vortex. Cells were then incubated at 37°C for 15 min and then 150 μl of freshly made, ice-cold methanol:acetic acid (3:1) were added cells in a drop-wise manner with agitation. Cells were pelleted and supernatants were removed until 0.5 ml media remained. Cells were resuspended in 5 ml of methanol:acetic acid added in a drop-wise manner under agitation. Cells were then fixed on ice for 20 min. Cells were washed once with methanol:acetic acid before being spotted onto slides. Slides were left to dry and immediately treated with pre-extraction buffer (0.1% TritonX-100, 20 mM HEPES-KOH pH 7.9, 50 mM NaCl, 3 mM MgCl2, 300 mM sucrose) for 10 min at RT and then washed with 0.5% TritonX-100 in phosphate buffered saline (PBS) for 10 min at RT. After permeabilization, cells were washed with PBS and treated with RNase H1 (50 U/ml, TAKRA, Japan) for 60 min at RT. Slides were rinsed with ice-cold PBS, fixed with 4% Paraformaldehyde (PFA), and finally fixed with ice-cold methanol for 30 min at RT. Slides were blocked with blocking buffer (PBS containing 4% BSA and 0.1% TritonX-100) for 1 h followed by incubation with primary antibodies against S9.6, m6A, RAD51, YTHDC1, pRPA2 S33, and GFP overnight at 4°C. For immunofluorescence co-localization with gH2AX and RAD51 staining were performed as described previously with minor modifications (7). Cells were fixed with 4 % paraformaldehyde for 20 min at RT. After microirradiation cells were washed immediately PBS and fixed with 4 % paraformaldehyde for 20 min at RT. All fixed cells were rinsed PBS two time, permeabilized with 0.5 % Triton X-100 in PBS for 5 min at RT. Cells were then washed twice in PBS and incubated with blocking buffer (4% BSA in PBS) for 1 hr at RT followed by incubation with primary antibody for γH2AX, Rad51, and m6A for overnight at 4 °C. Slides and cells were washed three times with wash buffer (PBS containing 0.1% TritonX-100) and subsequently incubated with secondary antibodies conjugated with fluorophores (Alexa Fluor 594 or Alexa Fluor 488, Thermo Fisher Scientific) and 4,6-diamidino-2-phenylindole (DAPI, 1 μg/ml, Thermo Fisher Scientific) for 1 h at RT. After three washes with wash buffer, slides were mounted using VectaMount AQ Aqueous Mounting Medium (Vector Laboratories, Burlingame, CA). Images were captured and analyzed using a LSM-880 confocal microscope. Nuclear fluorescence intensities were quantified in each sample using ZEN Blue software (Carl Zeiss). Numbers above each sample indicate n values, which are the numbers of nuclei analyzed. Cells with ≥10 overlapping S9.6 and m6A foci were counted as positive and the proportion of positive cells among all cells was determined. More than 100 cells were examined for each sample.

**EdU incorporation**

For EdU incorporation analysis, transfected cells were treated with EdU (10 µM) for 60 min before harvesting and fixing with methanol:acetic acid before being spotted onto slides. Cells were permeabilized with PBS containing 0.5% Triton X-100 for 10 min at room temperature. The click-it reaction was performed according to the manufacturer’s instructions. Slides were blocked with blocking buffer for 1 h followed for further immunostaining.

**Immunoprecipitation and immunoblotting**

For immunoprecipitation, cells were washed with ice-cold PBS and then lysed in NETN buffer (0.5% Nonidet P-40, 20 mM Tris [pH 8.0], 50 mM NaCl, 50 mM NaF, 100 µM Na3VO4, 1 mM dithiothreitol [DTT], and 50 µg/ml phenylmethylsulfonyl fluoride [PMSF]) with benzonase (Enzynomics, M018H) at 4°C for 40 min. Crude lysates were cleared by centrifugation at 14,000 rpm at 4°C for 5 min and supernatants were incubated with protein A-agarose-conjugated primary antibodies, FLAG-M2 affinity gel (Sigma, Cat#A2220), or c-Myc agarose affinity gel (Sigma, Cat#7470). Immunocomplexes were washed three times with NETN buffer and then subjected to sodium dodecyl sulfate-polyacrylamide gel electrophoresis (SDS-PAGE). Western blotting was performed using the antibodies indicated in figure legends. Proteins were visualized using secondary horseradish peroxidase-conjugated antibodies (Enzo Life Sciences, New York, NY) and enhanced chemiluminescence reagent (Thermo Fisher Scientific). Signals were detected using an automated imaging system (ChemiDoc™; Bio-Rad Laboratories, Hercules, CA).

**Antibodies**

The following antibodies were used in the present study: anti-Flag-HRP (Sigma, A8592), anti-Myc-HRP antibody (Roche, 11814150001), anti-GFP (Clontech, 632380), anti-β-actin antibody (Sigma, A5441), Anti-γH2AX antibodies (Cell signal, #2577, Sigma, 05636), anti-DDX41 antibody (Abnova, H00051428-M01, Altas antibodies, HPA049807), anti-METTL3 antibody (Abclonal, A8370), anti-METTL14 antibody (Sigma-Aldrich, HPA038002), anti-YTHDC1 antibody (Abcam, ab122340), anti-S9.6 antibody (Kerafast, ENH001), anti-m6A antibody (Synaptic Systems, 202003), anti-ATR antibody (Bethyl Laboratories, A300-137A), anti-phosphoATR antibody (S428, Cell signaling, #2853), anti-CHK1(Santa Cruz, SC-8408), anti-phosphoCHK1 (S317, Cell Signaling, #2344), anti-phosphoCHK1 (S345, Cell Signaling, #2341), anti-RPA2 antibody (Bethyl Laboratories, A300-244A), anti-phosphoRPA2 (S33, Bethyl Laboratories, A300-246A), anti-THOC1 antibody (Abcam, ab487), anti-FTO antibody (Abcam, ab126605), anti-BLM antibody (Abcam, ab2179), anti-FANCD2 antibody (Novus Biologicals, NB100-182), RAD51 antibody (Abcam, ab3801), and horseradish peroxidase-conjugated secondary antibodies specific to rabbit (Sigma-Aldrich, A0545) or mouse (Sigma-Aldrich, A9917) IgG.

**Laser micro-irradiation and cell imaging**

For laser microirradiation, cells were grown on 35 mm glass bottom dishes (SPL, Korea). Hela cells were transfected with plasmids or siRNA for 24 h. Transfected cells were treated with 10 μM BrdU (Sigma-Aldrich) prior to laser microirradiation for 20 h. Laser-scanning confocal microscopy was performed using a Zeiss LSM880 microscope with a 40x W (N.A. 1.2) C-apo objective. DNA damage was induced in live cells (maintained at 37°C in a humidified environment at 5% CO_2_) using a 355-nm UVA optically pumped semiconductor laser (Coherent, Genesis, 15 µm/s, 100% power, 20 iterations). For GFP-tagged proteins, time-lapse images were acquired at 10 s time intervals after laser microirradiation. Acquisition and analysis were performed using ZEN software (black edition, Zeiss, Germany).

**Cell proliferation assays**

5 × 10^4^ of *DDX41* KO or KI cells were plated in 6-well plates. The number of living and dead cells was evaluated by Trypan Blue exclusion every 24 h for 120 h as previously described (7). Cells were then counted every 24 h using a hemocytometer. Each experiment was repeated three times.

**Purification of DDX41 constructs**

Full-length DDX41, DDX41_ΔHel (helicase c domain deletion), DDX41_ΔDEAD (DEAD domain deletion), DDX41_ΔHel-Y259C, and DDX41-R525H mutants, all of which contain triple FLAG residues at the N-terminus and ten His residues at the C-terminus, were subcloned into pET19b-derived plasmid vectors. Each construct was cultured in Rosetta (DE3) *E. coli* strain (Millipore, 70954; 20 L culture for full-length DDX41 and 10 L culture for others) with 0.1 mg/mL carbenicillin until OD_600_ reached approximately 0.6. Plasmid expression was induced by the addition of 1 mM IPTG. Cells were further incubated at 16°C for 16 hrs. Harvested cells were resuspended in 200 mL lysis buffer (25 mM Tris-HCl [7.5], 100 mM NaCl, 10% glycerol, 0.5 mM EDTA, 1 mM DTT, 1 mM PMSF, and 1x Halt protease inhibitor [Thermofisher, 31170724-2]) and then lysed by sonication. Cell lysates were clarified by centrifugation at 30,000 g for 40 min at 4°C. For full-length DDX41, 15 mL of clarified lysates were loaded onto a Talon gravity column (Clontech, 635503) equilibrated with Talon A buffer (50 mM HEPES [7.5], 200 mM NaCl, and 30 mM imidazole). The column was then washed with 100 mL Talon A buffer. Proteins were eluted with Talon B buffer (50 mM HEPES [7.5], 200 mM NaCl, and 500 mM imidazole). In the ÄKTA pure FPLC system (Cytiva), 1 mL of HiTrap Q HP column (Cytiva, 17-1154-01) was equilibrated with QA buffer (25 mM Tris-HCl [7.5], 10% glycerol, 0.5 mM EDTA, and 1 mM DTT). Pooled fractions from the Talon column were loaded onto a Q HP column which was then washed with 100 mL of 10% QB buffer (25 mM Tris-HCl [7.5], 1 M NaCl, 10% glycerol, 0.5 mM EDTA, and 1 mM DTT). Full-length DDX41 proteins were eluted using linear gradient from 10% to 100% QB buffer. Eluates were collected in 1 mL fractions. For DDX41_ΔHel (helicase c domain deletion), DDX41_ΔHel Y259C, and DDX41_ΔHel R525H, 30 mL of clarified cell lysate was loaded onto a MHAP column (Bio-Rad, 1570020) equilibrated with MHAP A buffer (25 mM Tris-HCl [7.5], 100 mM NaCl, 10% glycerol, 0.5 mM EDTA, and 1 mM DTT) using the ÄKTA pure FPLC system (Cytiva). The MHAP column was then washed with 100 mL of 10% MHAP B buffer (25 mM Tris-HCl, 100 mM NaCl, 10% glycerol, 0.5 mM EDTA, 1 mM DTT, and 1 M KH_2_PO_4_; pH adjusted to 6.3 with NaOH). DDX41 constructs were eluted using linear gradient from 10% to 80% MHAP B buffer. Eluates were collected in 5 mL fractions and target protein-containing fractions were pooled. 15 mL of pooled fractions were loaded onto a Talon column equilibrated with Talon A buffer. The column was then washed with 100 mL of Talon A buffer. Proteins were eluted with Talon B buffer. All purified proteins were finally dialyzed against storage buffer (25 mM HEPES [7.5], 300 mM NaCl, 1 mM DTT, 10% glycerol, and 1 mM EDTA) and then snap-frozen in liquid nitrogen for storage at -80°C.

**Electrophoretic mobility shift assay** (**EMSA)**

The binding affinity of DDX41 constructs with diverse substrates were measured using EMSA. Various oligomers (Supplementary Table 4) were synthesized (Bioneer, South Korea) and diluted in DDX41 reaction buffer (25 mM Tris-HCl [8.0], 100 mM NaCl) as described in Supplementary Table 5. For annealing, each pair of oligomers was mixed and heated to 95°C followed by slow cooling to 23°C. 10 nM of each annealed DNA substrate was incubated with several types of DDX41 constructs at different concentrations (0, 80, 160, 325, 650, and 1300 nM) in DDX41 reaction buffer in a dark room at 23°C for 30 min. Reactants were run on 5% non-denaturing PAGE in 0.5x TBE and fluorescence signals were measured using Typhoon 2000 imager (Cytiva). The binding ratio was calculated by dividing the intensity of shifted bands by the sum of shifted and non-shifted bands using ImageJ (NIH).

**Homologous recombination assay**

The homologous recombination assay was performed as described previously with some adaptations (7). U2OS DR-GFP cells were transfected with the DDX41 siRNAs in 6 well plates, and after 24 h, they were transfected with mCherry empty vector and I-SceI expression vector. Thirty-six hours after I-SceI transfection, cells were trypsinized, and the percentages of mCherry/GFP double positive cells were determined by flow cytometry.

**Whole-genome sequencing library construction and sequencing**

100 ng of genomic DNA for a 350 bp insert size was fragmented using a Covaris S2 Ultrasonicator. DNA sequencing libraries were constructed using the TruSeq Nano DNA Sample Preparation Kit from Illumina (San Diego, CA) according to the manufacturer protocol. The qualities of amplified libraries were confirmed by electrophoresis on Agilent Bioanalyzer High Sensitivity DNA Kit (part # 5067-4626) (Agilent, CA). Libraries were quantified using the KAPA Library Quantification Kit (KK4824) (Kapa Biosystems, MA) according to the manufacturer’s library quantification protocol. Following cluster amplification of denatured templates, sequencing was progressed as paired-end (2×150bp) using Illumina Novaseq6000 (Illumina).

**Whole-genome sequencing alignment**

The Burrows–Wheeler Aligner MEM (v.0.7.17) (8) tool was conducted to align whole-genome sequencing reads with the human reference genome GRCh38/hg38. We used the GATK (v.4.1.9.0) pipeline (9), including MarkDuplicates, BaseRecalibrator, and ApplyBQSR, for the pre-processing of raw aligned reads to correct technical biases.

**Single nucleotide variant and short insertion and deletion detection**

Mutect2 (10) was performed to identify single nucleotide variants (SNVs) and short insertions and deletions (indels) in nine subject samples with a WT sample as control. To remove alignment artifacts and get somatic variants, the GATK Mutect2 workflow was applied, including CalculateContamination, LearnReadOrientationModel and FilterMutectCalls. Additional filtering was employed to eliminate false positive calls. SNVs and indels were required to have a variant allele fraction of at least 0.4 and at least twelve variant-supporting reads. SNVs and indels, a read depth more than 15 or less than 150 at the variant site, were only used. We only analyzed the variants that have at least one read supporting them in each direction.

**Structural variant identification**

In order to identify structural variations (SVs), Delly2 (v.0.8.7) (11) was employed. By comparing the samples with a WT control sample, SVs were detected. We applied the default Delly2 quality filter (mapping quality should be greater than 20 and paired-end support reads should be greater than five for translocations or three for other SVs). Deletions and insertions longer than 200bp were only used. SVs were required to have at least ten variant-supporting reads.

**Statistical analyses**

All results are presented as the mean ± standard deviation of the determinations. Comparisons between groups were performed using one-way or two-way ANOVA. P-values less than 0.05 were considered statistically significant. All statistical analyses were performed using Prism 10.0.0 (GraphPad). R software (v.4.0.3)(12) was used to perform the statistical analyses. For the t-test, the ggpubr package's (13) compare_means function was employed. The supplementary table provides the number of samples and variant utilized in the analysis.

**Supplementary materials and methods references**

1. Richards S, Aziz N, Bale S, Bick D, Das S, Gastier-Foster J, et al. Committee ALQA (2015) Standards and guidelines for the interpretation of sequence variants: a joint consensus recommendation of the American College of Medical Genetics and Genomics and the Association for Molecular Pathology. Genetics in Medicine. 2015;17(5):405-24.

2. Li MM, Datto M, Duncavage EJ, Kulkarni S, Lindeman NI, Roy S, et al. Standards and guidelines for the interpretation and reporting of sequence variants in cancer: a joint consensus recommendation of the Association for Molecular Pathology, American Society of Clinical Oncology, and College of American Pathologists. The Journal of molecular diagnostics. 2017;19(1):4-23.

3. Churpek JE, Smith-Simmer K. DDX41-Associated Familial Myelodysplastic Syndrome and Acute Myeloid Leukemia. Adam MP, Everman DB, Mirzaa GM, Pagon RA, Wallace SE, Bean LJH, et al., editors. Seattle (WA): University of Washington, Seattle

Copyright © 1993-2022, University of Washington, Seattle. GeneReviews is a registered trademark of the University of Washington, Seattle. All rights reserved.; 2021.

4. Qu S, Li B, Qin T, Xu Z, Pan L, Hu N, et al. Molecular and clinical features of myeloid neoplasms with somatic DDX41 mutations. British Journal of Haematology. 2021;192(6):1006-10.

5. Shin WY, Yoon SY, Park R, Kim J-A, Song HH, Bang HI, et al. A novel bi-alleleic DDX41 mutations in B-cell lymphoblastic leukemia: case report. BMC Medical Genomics. 2022;15(1):1-7.

6. Nguyen HD, Yadav T, Giri S, Saez B, Graubert TA, Zou L. Functions of Replication Protein A as a Sensor of R Loops and a Regulator of RNaseH1. Molecular Cell. 2017;65(5):832-47 e4.

7. Ju MK, Shin KJ, Lee JR, Khim KW, E AL, Ra JS, et al. NSMF promotes the replication stress-induced DNA damage response for genome maintenance. Nucleic Acids Research. 2021;49(10):5605-22.

8. Li H. Aligning sequence reads, clone sequences and assembly contigs with BWA-MEM. arXiv preprint arXiv:13033997. 2013.

9. McKenna A, Hanna M, Banks E, Sivachenko A, Cibulskis K, Kernytsky A, et al. The Genome Analysis Toolkit: a MapReduce framework for analyzing next-generation DNA sequencing data. Genome research. 2010;20(9):1297-303.

10. Benjamin D, Sato T, Cibulskis K, Getz G, Stewart C, Lichtenstein L. Calling somatic SNVs and indels with Mutect2. BioRxiv. 2019:861054.

11. Rausch T, Zichner T, Schlattl A, Stütz AM, Benes V, Korbel JO. DELLY: structural variant discovery by integrated paired-end and split-read analysis. Bioinformatics. 2012;28(18):i333-i9.

12. R Core Team R. R: A language and environment for statistical computing. 2013.

13. Kassambara A. ggpubr: “ggplot2” based publication ready plots (Version 0.4.0). R package. 2020 [Available from: <https://cran.r-project.org/web/packages/ggpubr/index.html>.

**Supplementary Figures**

Supplementary Figure S1

**
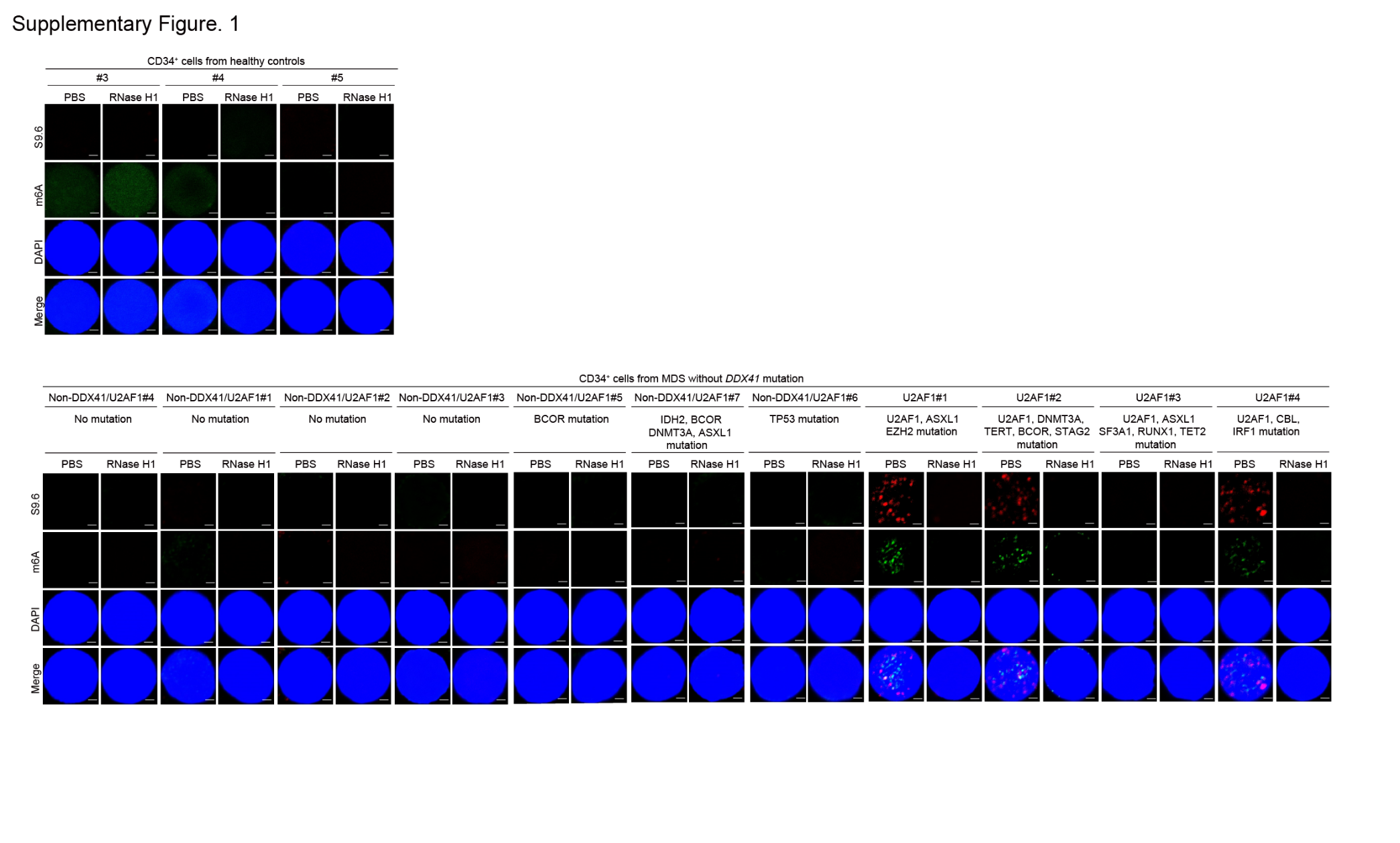
**

**Supplementary Figure S1. m6A and R-loop in CD34^+^ bone marrow cells from non-DDX41 mutated MDS and healthy controls.**

Representative immunofluorescence images of localization m6A and S9.6 in CD34^+^ cells isolated from MDS and healthy BM. Scale bar, 1 μm.

Supplementary Figure S2

**
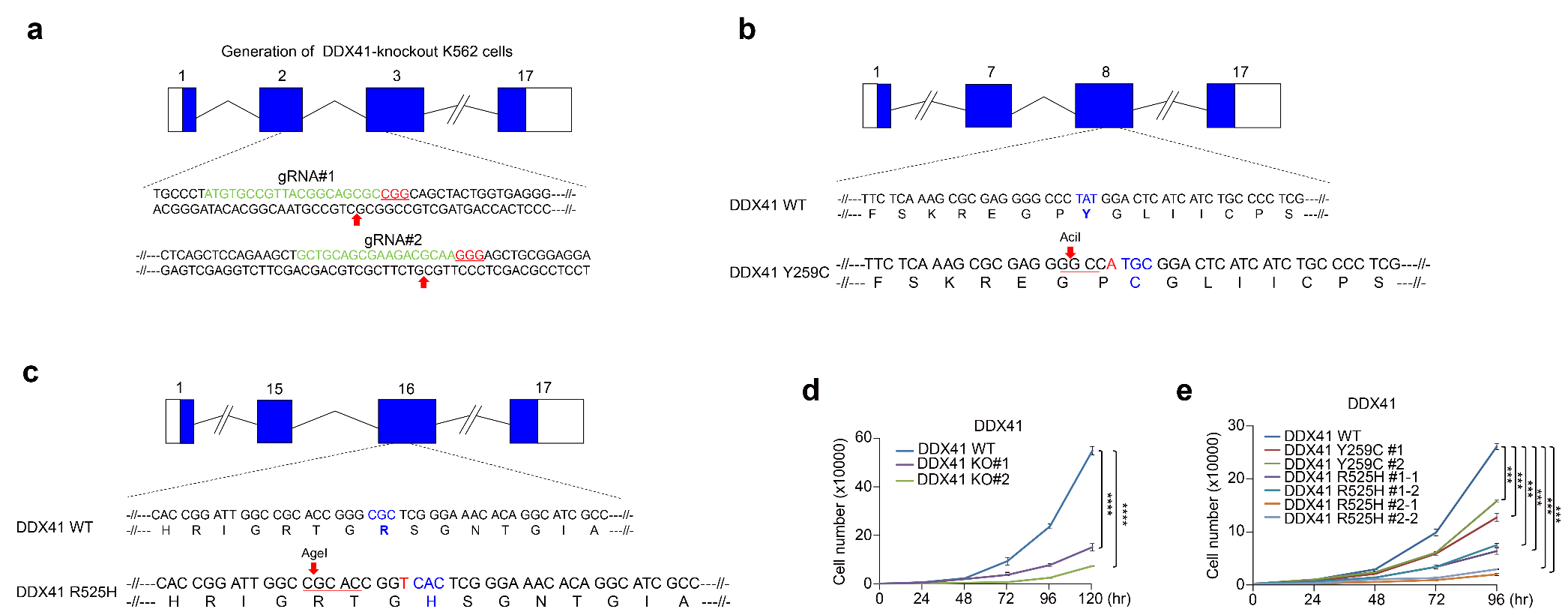
**

**Supplementary Figure S2. Generation of *DDX41* KO or KI K562 cell lines.**

**(a)** Human DDX41 genomic structure and guide RNAs in exon 2 and 3. Guide RNA plasmid for human DDX41 coupled with GFP vector was co-transfected to K562 cells using Lipofectamine 3000 reagent. 48 hr after transfection, GFP-positive K562 cells were sorted by FACS and then seeded onto 96-well plates. Each growing clones were expanded and screened for DDX41 knockout by western blotting and DNA sequencing. Red arrow: cut site, green: target sequence for guide RNA, Red (underlined): PAM sequence. **(b and c)** Human *DDX41* genomic structure and guide RNAs in exon 8 (Y259C, **b**) and 10 (R525H, **c**). **(d and e)** Cell proliferation of *DDX41* KO or KI K562 cell lines. 5000 cells were plated, and the number of viable cells was counted at indicated time points. Results are presented as the average of three independent experiments. Error bars indicate standard deviation. *P*-value was calculated based on two-way ANOVA in **(d, e)** (^***^*P* < 0.001, ^****^*P* < 0.0001).

Supplementary Figure S3

**
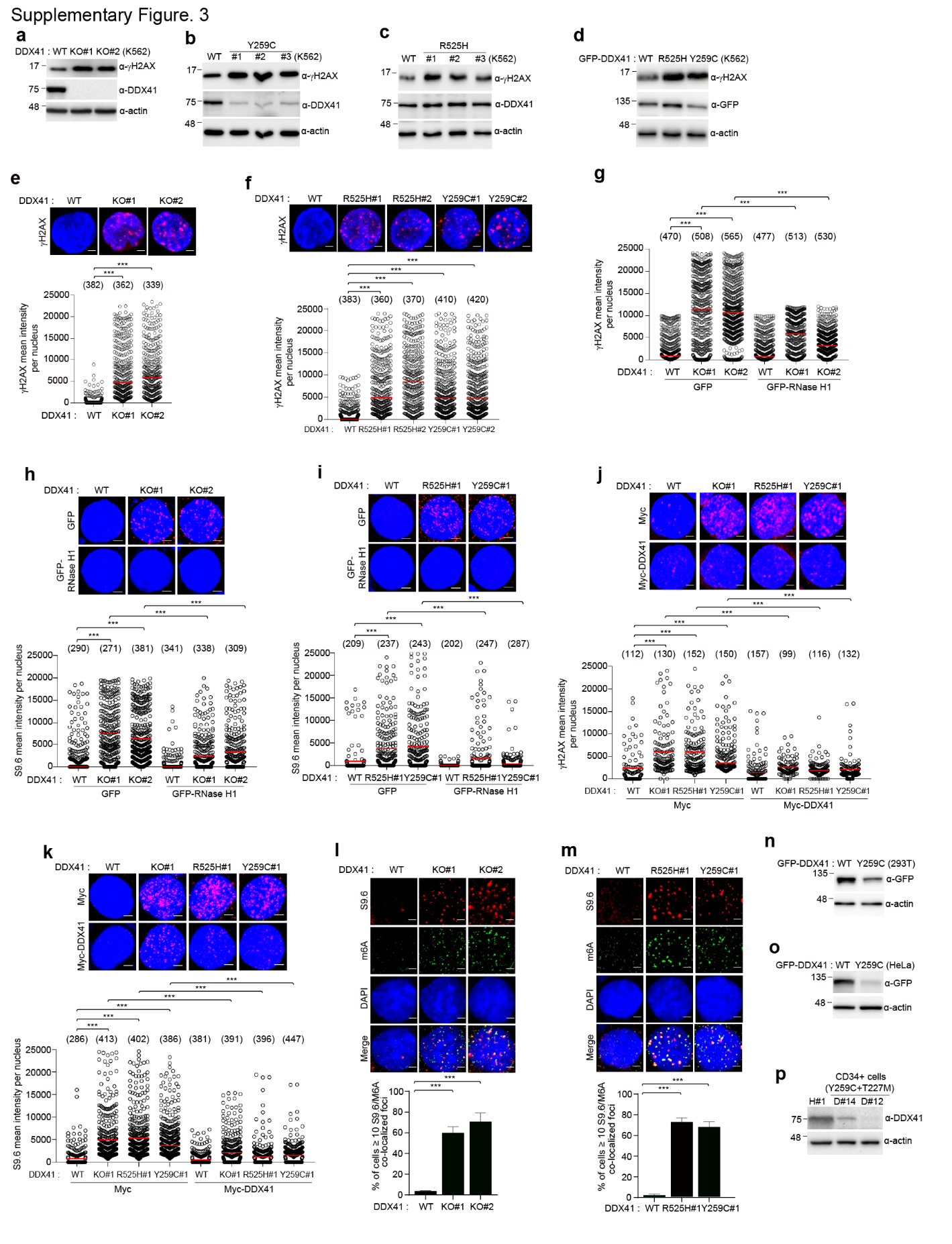
**

**Supplementary Figure S3.** **DNA damage accumulated through methylated-R-loop in *DDX41* KO or KI cell lines.**

**(a-c)** *DDX41* WT or KO **(a)** or KI **(b, c)** K562 cell lysates were immunoblotted with indicated antibodies. (**d)** DDX41 WT, R525H or Y259C expression transfected cell lysates were immunoblotted with indicated antibodies. **(e, f)** Quantification of γH2AX fluorescence intensity was determined by immunofluorescence staining with γH2AX antibodies in *DDX41* WT, KO, and KI K562 cells. **(g-i)** *DDX41* WT or KO or KI K562 cells were transfected with GFP or GFP-RNase H1 expression plasmid. After 48 hr, γH2AX **(g)**, and S9.6 **(h, i)** intensity were determined by immunofluorescence. **(j, k)** γH2AX **(j)** and S9.6 **(k)** intensity in *DDX41* WT, KO, and KI K562 cells were reduced by overexpressed Myc or Myc-DDX41. After 72 hr, γH2AX and S9.6 intensity were determined by immunofluorescence. The numbers above each sample indicate the n value, which is the number of nuclei analyzed. Scale bar, 5 μm. **(l, m)** Colocalization of S9.6 and m6A in *DDX41* KO **(l)**, and KI **(m)** K562 cells. Quantification of S9.6 and m6A fluorescence intensity was determined by immunofluorescence staining with S9.6 and m6A antibodies in *DDX41* WT, KO, and KI K562 cells. Results are presented as the average of three independent experiments. Error bars indicate standard deviation. Scale bar, 5 μm. **(n-p)** Cell lysates of GFP-*DDX41* WT or Y259C transfected HEK293T **(n)**, HeLa **(o)**, or CD34^+^ cells from healthy or Y259C mutant MDS patients were immunoblotted with indicated antibodies. *P*-value was calculated based on one-way ANOVA in **(e-m)** (^***^*P* < 0.001).

Supplementary Figure S4

**
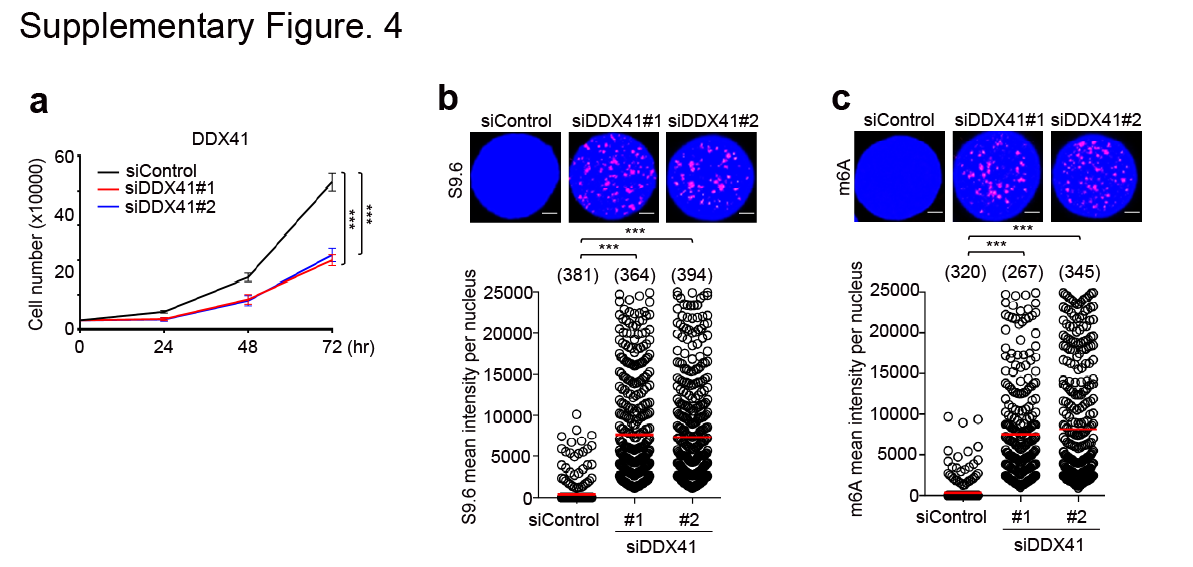
**

**Supplementary Figure S4. Reduced cell proliferation and accumulated S9.6 and m6A signals in SKM-1 cell lines.**

**(a)** Cell proliferation of control or two different *DDX41* siRNAs transfected SKM-1 cells. SKM-1 cells were transfected with control or two different *DDX41* siRNAs. After 48 h later, 5000 cells were plated, and the number of viable cells was counted at indicated time points. Results are presented as the average of three independent experiments. Error bars indicate standard deviation. **(b, c)** Quantification of S9.6 or m6A fluorescence intensity in control or two different *DDX41* siRNAs transfected SKM-1 cells. SKM-1 cells were transfected with control or two different *DDX41* siRNAs. After 48 h later, Quantification of S9.6 or m6A fluorescence was determined by immunofluorescence staining with S9.6 or m6A antibodies. Results are presented as the average of three independent experiments. Error bars indicate standard deviation. *P*-value was calculated based on two-way ANOVA in **(a)**, and one-way ANOVA in **(b-c)** (^***^*P* < 0.001). Scale bar, 5 μm.

Supplementary Figure S5

**
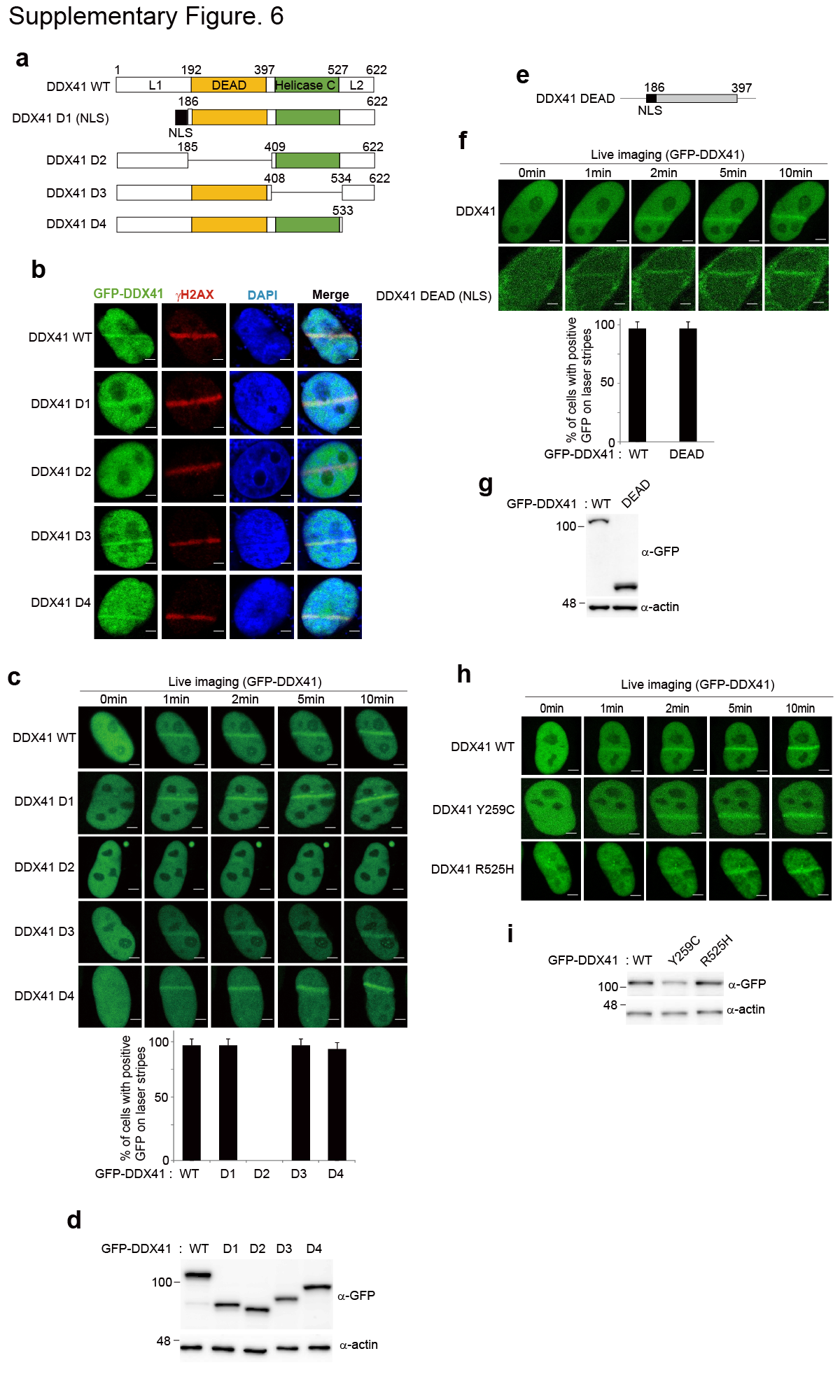
**

**Supplementary Figure S5. DDX41 localizes to DNA damage sites via the DEAE box of DDX41.**

**(a)** Schematic diagram of the DDX41 deletion mutants. **(b)** HeLa cells expressing GFP-DDX41 WT and deletion mutants were subjected to laser microirradiation. After 10 min, cells were fixed and stained with anti-GFP and anti-γH2AX antibodies. 4,6-diamidino-2-phenylindole (DAPI) was used to stain nuclei. Scale bar, 5 μm. (**c)** HeLa cells were transfected with GFP-DDX41 WT or deletion mutant expression plasmids, and 24 hrs later, the cells were treated with laser microirradiation (top panel). The cells with positive GFP expression on the laser stripes are presented in the bar graph (bottom panel). Scale bar, 5 μm. The results represent the average of two independent experiments. The error bars indicate the standard deviation for the cells transfected with each expression plasmid. **(d)** The expression validation of GFP-DDX41 WT and -DDX41 deletion mutants by Western blotting. **(e)** Diagram of the DDX41-DEAD mutant. The numbers indicate the amino acids residues. **(f)** GFP-DDX41-DEAD mutant translocates to the DNA damage sites. HeLa cells were transfected with GFP-tagged mutant expression plasmids, and 24 hrs later, the cells were treated with laser microirradiation (top panel). Scale bar, 5 μm. The cells with positive GFP expression on the laser stripes are presented in the bar graph (bottom panel). The results represent the average of two independent experiments. The error bars indicate the standard deviation for the cells transfected with each expression plasmid. **(g)** The expression validation of GFP-DDX41 WT or -DDX41-DEAD mutants by western blotting. **(h)** HeLa cells were transfected with GFP-DDX41 WT, -DDX41 Y259C and -DDX41 R525H expression plasmids, and 24 hrs later, the cells were treated with laser microirradiation. Scale bar, 5 μm. **(i)** The expression validation of GFP-DDX41 WT, -DDX41 Y259C and -DDX41 R525H mutants by western blotting.

Supplementary Figure S6

**
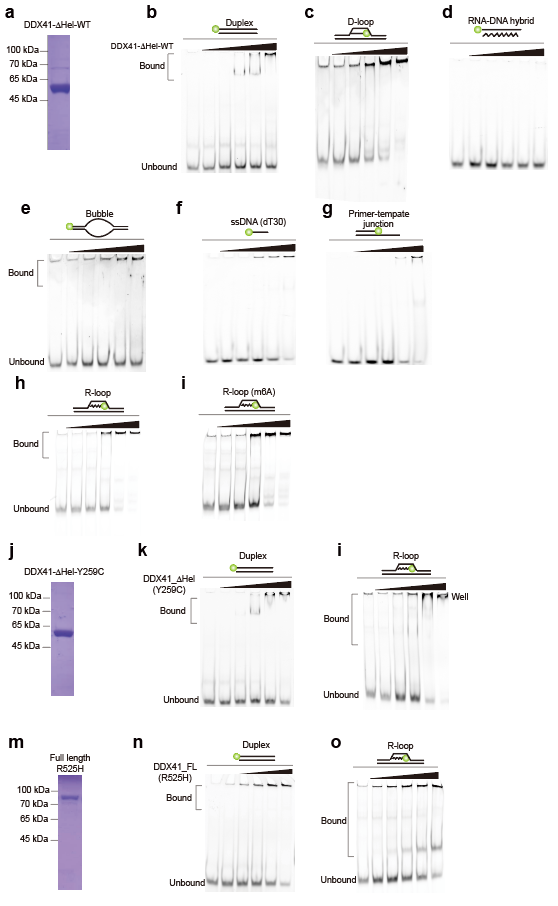
**

**Supplementary Figure S6.** **Binding affinity of various construct of DDX41 to nucleotide substrates.**

**(a)** SDS PAGE data of purified recombinant DDX41-ΔHel-WT. **(b-i)** EMSA for DDX41-ΔHel-WT and Duplex **(b)**, D-loop **(c)**, RNA-DNA hybrid **(d**), Bubble **(e)**, ssDNA(dT30) **(f)**, Primer-template junction **(g)**, R-loop **(h)**, methylated R-loop **(i)**. DDX41-ΔHel-WT (0, 80, 160, 325, 650 and 1300 nM) was titrated to 10 nM of nucleotide substrates. **(j)** SDS PAGE data of purified recombinant DDX41-ΔHel-Y259C. **(k, l)** EMSA for DDX41-ΔHel-Y259C and Duplex **(k)**, R-loop **(l)**. DDX41-ΔHel-Y259C (0, 80, 160, 325, 650 and 1300 nM) was titrated to 10 nM of nucleotide substrates. **(m)** SDS PAGE data of purified recombinant DDX41-R525H. **(n, o)** EMSA for DDX41-ΔHel-Y259C and Duplex **(n)**, R-loop **(o)**. DDX41-R525H (0, 80, 160, 325, 650 and 1300 nM) was titrated to 10 nM of nucleotide substrates.

Supplementary Figure S7

**

**

**Supplementary Figure S7. DDX41 associates with METTL3, METTL14 and YTHDC1.**

**(a)** Diagram of DDX41 WT and deletion mutants (A1 to A4, N, and C). Numbers indicate amino acid residues. **(b-d)** WT SFB-METTL3, SFB-METTL14, or SFB-YTHDC1 and either Myc-DDX41 or corresponding deletion mutants were co-transfected into K562 cells. Cell lysates were immunoprecipitated with anti-Myc antibodies and then immunoblotted with indicated antibodies. **(e)** Diagram of WT METTL3 and deletion mutants (D1–D3). Numbers indicate amino acid residues. **(g)** Diagram of WT METTL14 and deletion mutants (D1 to D3). Numbers indicate amino acid residues. **(i)** Diagram of WT YTHDC1 and deletion mutants (D1–D7). Numbers indicate amino acid residues. Numbers indicate amino acid residues. **(f, h, j)** Interactions between overexpressed DDX41 and METTL3, METTL14 or YTHDC1. **(f)** Myc-DDX41 and either WT SFB-METTL3 or corresponding deletion mutants were co-transfected into K562 cells. **(h)** Myc-DDX41 and either WT SFB-METTL14 or corresponding deletion mutants were co-transfected into K562 cells. **(j)** Myc-DDX41 and either WT SFB-YTHDC1 or corresponding deletion mutants were co-transfected into K562 cells. Cell lysates were immunoprecipitated with anti-Myc antibodies and then immunoblotted with Flag antibodies. **(k)** Diagram of WT METLL3 and deletion mutants (METTL3 D4). The numbers indicate the amino acid residues. **(l)** Myc-DDX41 WT and either SFB-METTL3 or its deletion mutant were co-transfected into K562 cells. The cell lysates were immunoprecipitated with the anti-Flag antibody and then immunoblotted with the indicated antibodies. **(m)** Diagram of WT METLL14 and deletion mutants (METTL14 D4). The numbers indicate the amino acid residues. **(n)** Myc-DDX41 WT and either SFB-METTL14 or its deletion mutant were co-transfected into 293T cells. The cell lysates were immunoprecipitated with the anti-Flag antibody and then immunoblotted with the indicated antibodies. **(o)** Diagram of WT YTHDC1 and deletion mutant (YTHDC1 A4). The numbers indicate the amino acid residues. **(p)** SFB-DDX41 WT and either Myc-YTHDC1 or its deletion mutant were co-transfected into 293T cells. The cell lysates were immunoprecipitated with the anti-Flag antibody and then immunoblotted with the indicated antibodies.

Supplementary Figure S8

**
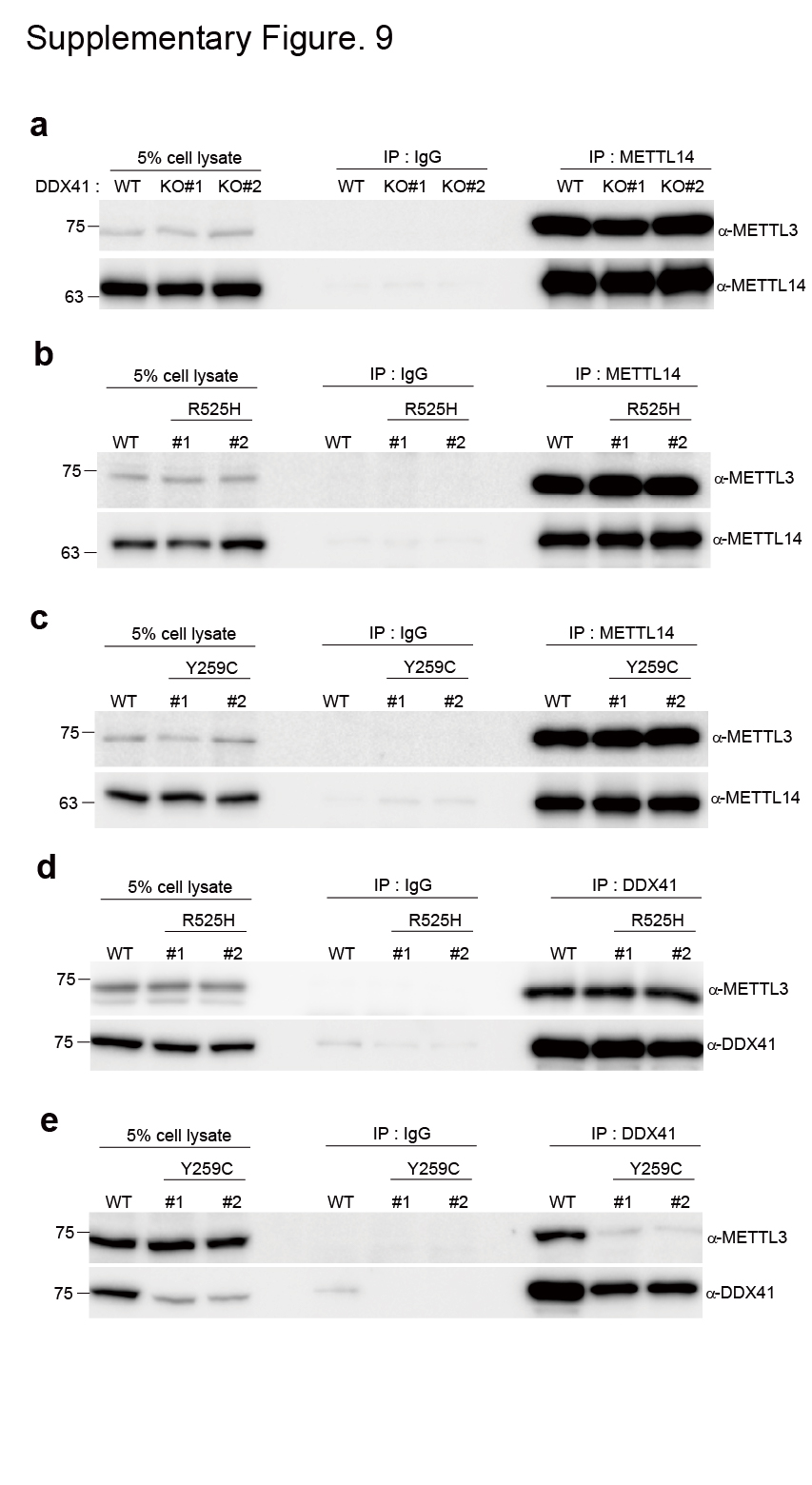
**

**Supplementary Figure S8. DDX41 mediates the interaction among DDX41, METTL3 and METTL14.**

**(a-c)** The interaction between METTL3 and METTL14 in *DDX41* KO or KI K562 cells. Immunoprecipitation reactions were performed using rabbit IgG or indicated antibodies and subjected to Western blotting analysis using the indicated antibodies. **(d, e)** The interaction between DDX41 and METTL3 in KI K562 cells. Immunoprecipitation reactions were performed using rabbit IgG or indicated antibodies and subjected to Western blotting analysis using the indicated antibodies.

Supplementary Figure S9

**
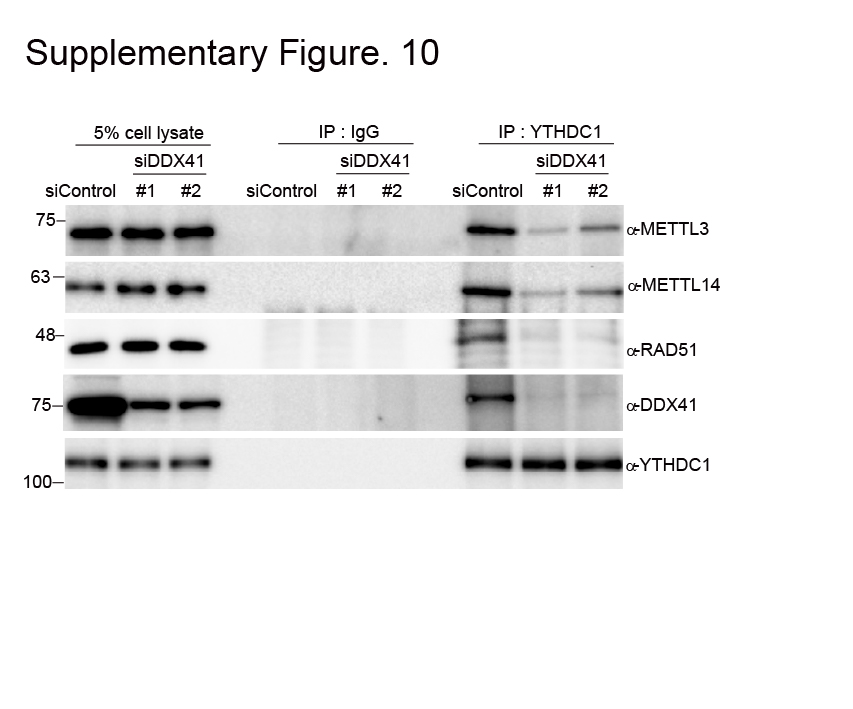
**

**Supplementary Figure S9. DDX41 mediates the interaction among DDX41, METTL3, RAD51 and METTL14 in SKM-1 cells.**

Immunoprecipitation reactions were performed using rabbit IgG or indicated antibodies and subjected to Western blotting analysis using the indicated antibodies.

Supplementary Figure S10

**
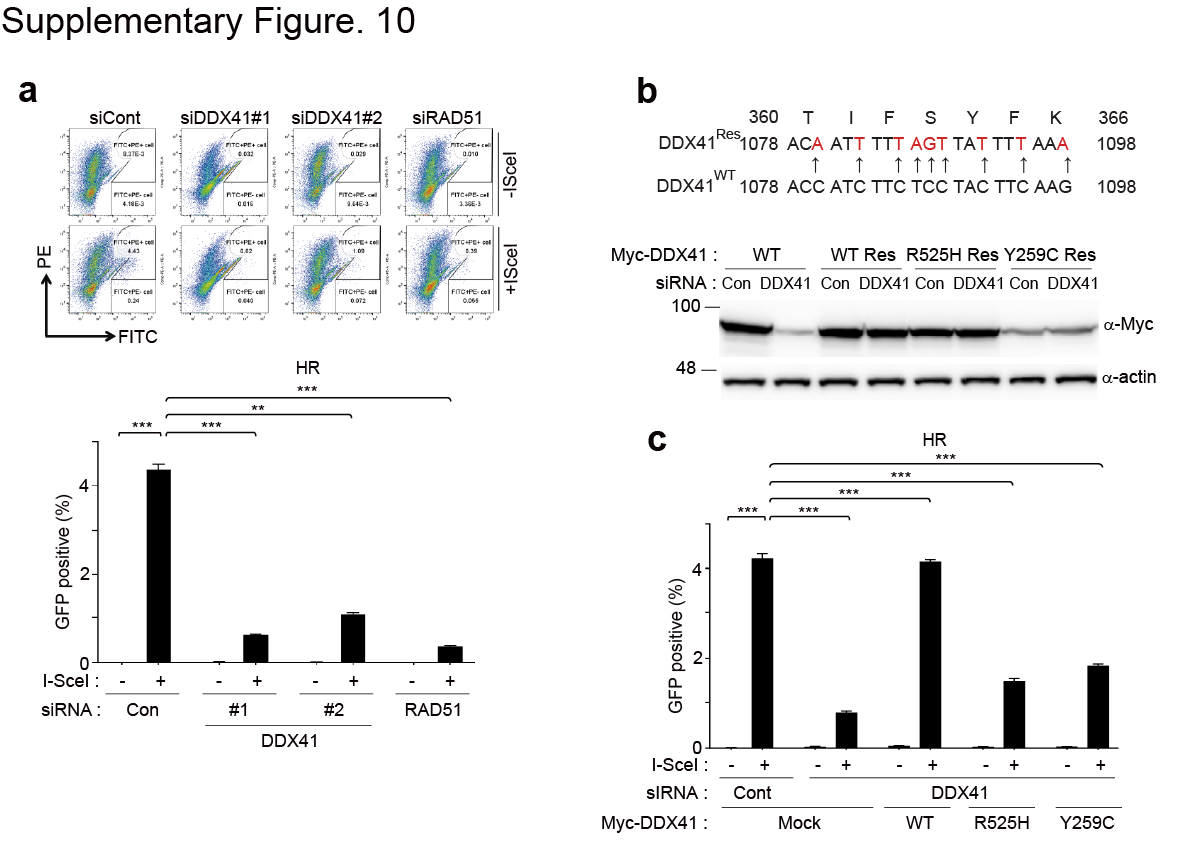
**

**Supplementary Figure S10. DDX41 is positive effector in homologous repair and generated the siRNA resistant DDX41expression DNA constructs**.

**(a)** Measurement of homologous recombination capacity in DR-GFP reporter U2OS cells. U2OS cells harboring the DR-GFP reporter were treated with the indicated siRNAs. Two days later, GFP expression was accessed by flow cytometry. The results represent the average of three independent experiments. The error bars indicate the standard deviation. **(b)** The sequences of the siRNA-resistant DDX41 WT cDNA showing the silent mutation (DDX41-RES). For protein expression of DDX41-RES, HEK293T cells were transfected siRNA for 24 hr and were transfected DDX41 plasmid as indicated for 24 hr. And then whole cell lysates were prepared for western blotting to confirm that the DDX41-RES is resistant to the DDX41 siRNA treatment. **(c)** Measurement of homologous recombination capacity in DR-GFP reporter U2OS cells. U2OS cells harboring the DR-GFP reporter were treated with the indicated siRNAs, followed by transfection with the indicated expression plasmids. Two days later, GFP expression was accessed by flow cytometry. The results represent the average of three independent experiments. The error bars indicate the standard deviation. *P*-value was calculated based on one-way ANOVA in **(a, c)** (^**^*P* < 0.01, ^***^*P* < 0.001).

Supplementary Figure S11

**
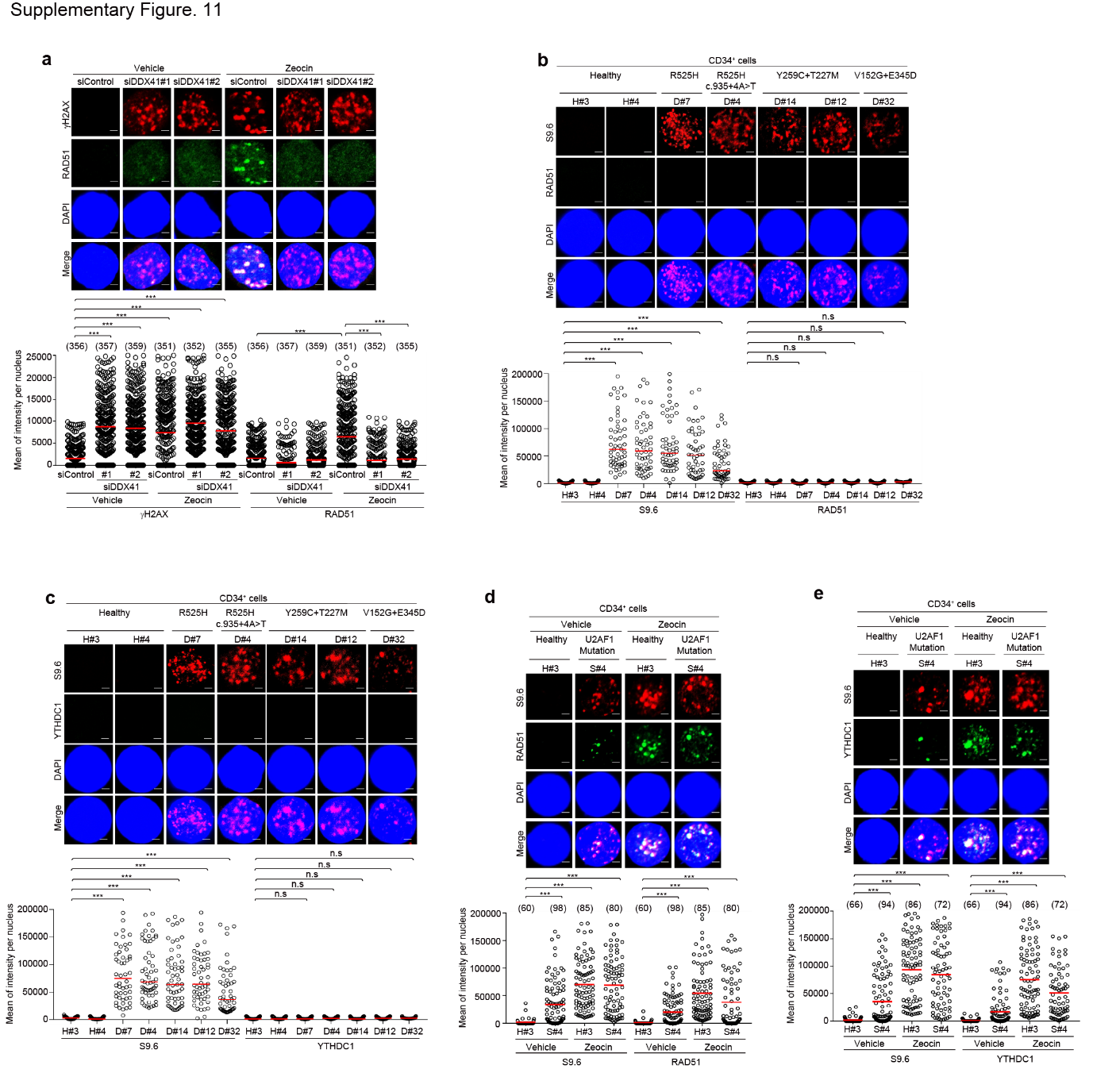
**

**Supplementary Figure S11. DDX41 recruits YTHDC1 and RAD51 to DNA damage sites.**

**(a)** Quantification of RAD51/γH2AX fluorescence intensity in control or two different *DDX41* siRNAs transfected SKM-1 cells. SKM-1 cells were transfected with control or two different *DDX41* siRNAs for 48 hr. And then the transfected SKM-1 cells were treated with Vehicle (DMSO) or Zeocin. After 12 hrs, the cells were with fixed and stained with indicated antibodies. Results are presented as the average of three independent experiments. Error bars indicate standard deviation. Scale bar, 5 μm. **(b, c)** S9.6/RAD51/YTHDC1 fluorescence intensities in CD34^+^ cells isolated from the BM of healthy controls or *DDX41* mutant MDS patients. CD34^+^ cells were fixed and S9.6/RAD51 **(b)** and S9.6/YTHDC1 **(c)** fluorescence intensities were determined by immunofluorescence. We counted 50 cells from healthy controls and patients. Scale bar, 1 μm. **(d, e)** S9.6/RAD51/YTHDC1 fluorescence intensities in CD34^+^ cells isolated from the BM of healthy controls or *U2AF1* MDS patients. CD34^+^ cells were fixed and S9.6/RAD51 **(d)** and S9.6/YTHDC1 **(e)** fluorescence intensities were determined by immunofluorescence. The numbers above each sample indicate the n value, which is the number of nuclei analyzed. Scale bar, 1 μm. *P*-value was calculated based on one-way ANOVA in **(a-e)** (^***^*P* < 0.001).

Supplementary Figure S12

**
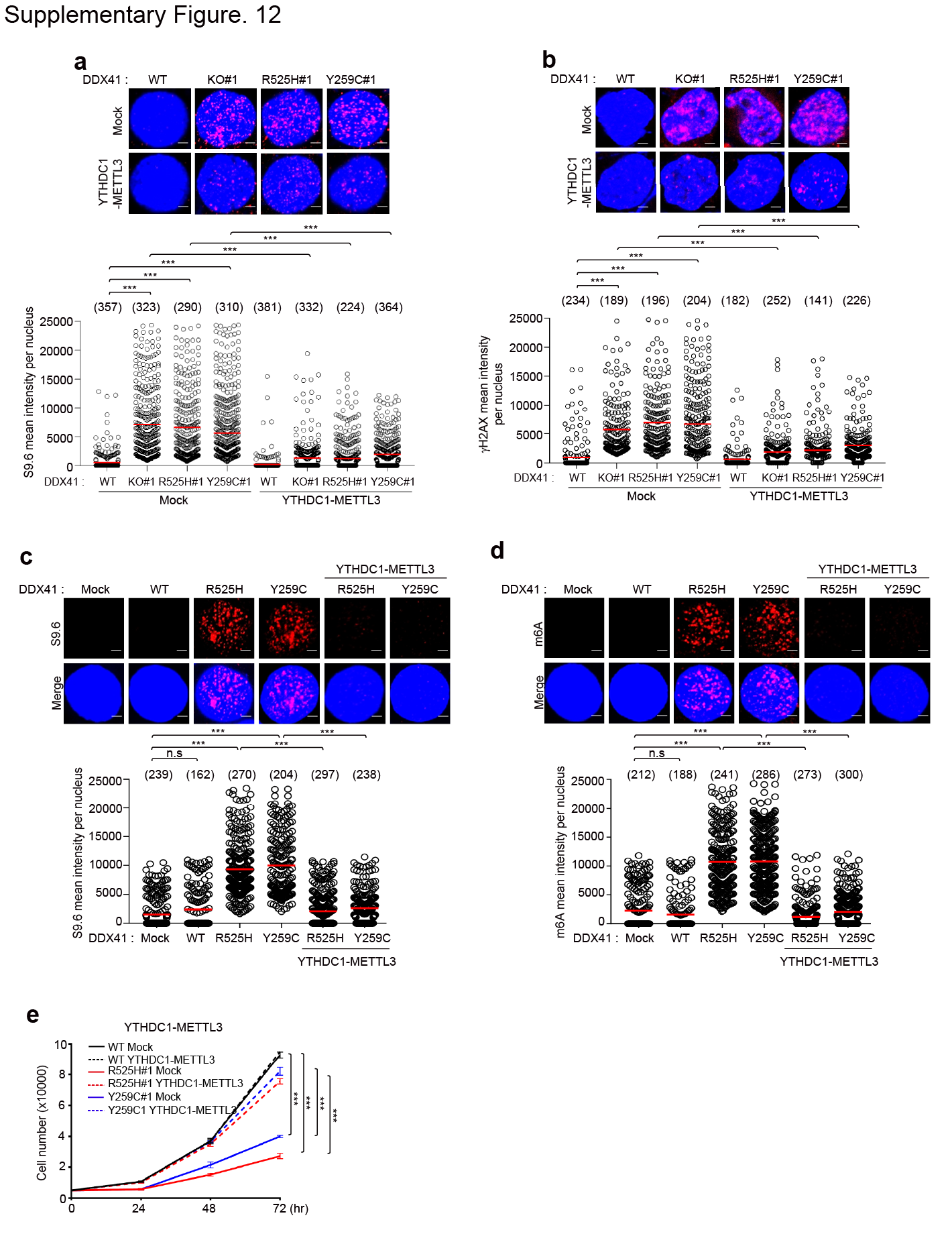
**

**Supplementary Figure S12. METTL3-YTHDC1 fusion protein rescues the DNA damage in *DDX41* dysfunctional cells.**

**(a, b)** *DDX41* WT, KO, and KI K562 cells were transfected with Mock or YTHDC1-METTL3 expression plasmids. After 72 hrs, S9.6 **(a)** and γH2AX **(b)** fluorescence intensities were determined by immunofluorescence**.** The numbers above each sample indicate the n value, which is the number of nuclei analyzed. Scale bar, 5 μm. **(c, d)** SKM-1 cells were transfected with indicated expression plasmids. After 72 hrs, S9.6 **(c)** and m6A **(d)** fluorescence intensities were determined by immunofluorescence**.** The numbers above each sample indicate the n value, which is the number of nuclei analyzed. Scale bar, 5 μm. **(e)** Viability of indicated plasmid transfected SKM-1 cells. 5000 cells were plated, and the number of viable cells was counted at indicated time points. Results are presented as the average of three independent experiments. Error bars indicate standard deviation. Scale bar, 1 μm. *P*-value was calculated based on one-way ANOVA in **(a-e)** (^***^*P* < 0.001).

Supplementary Figure S13

**
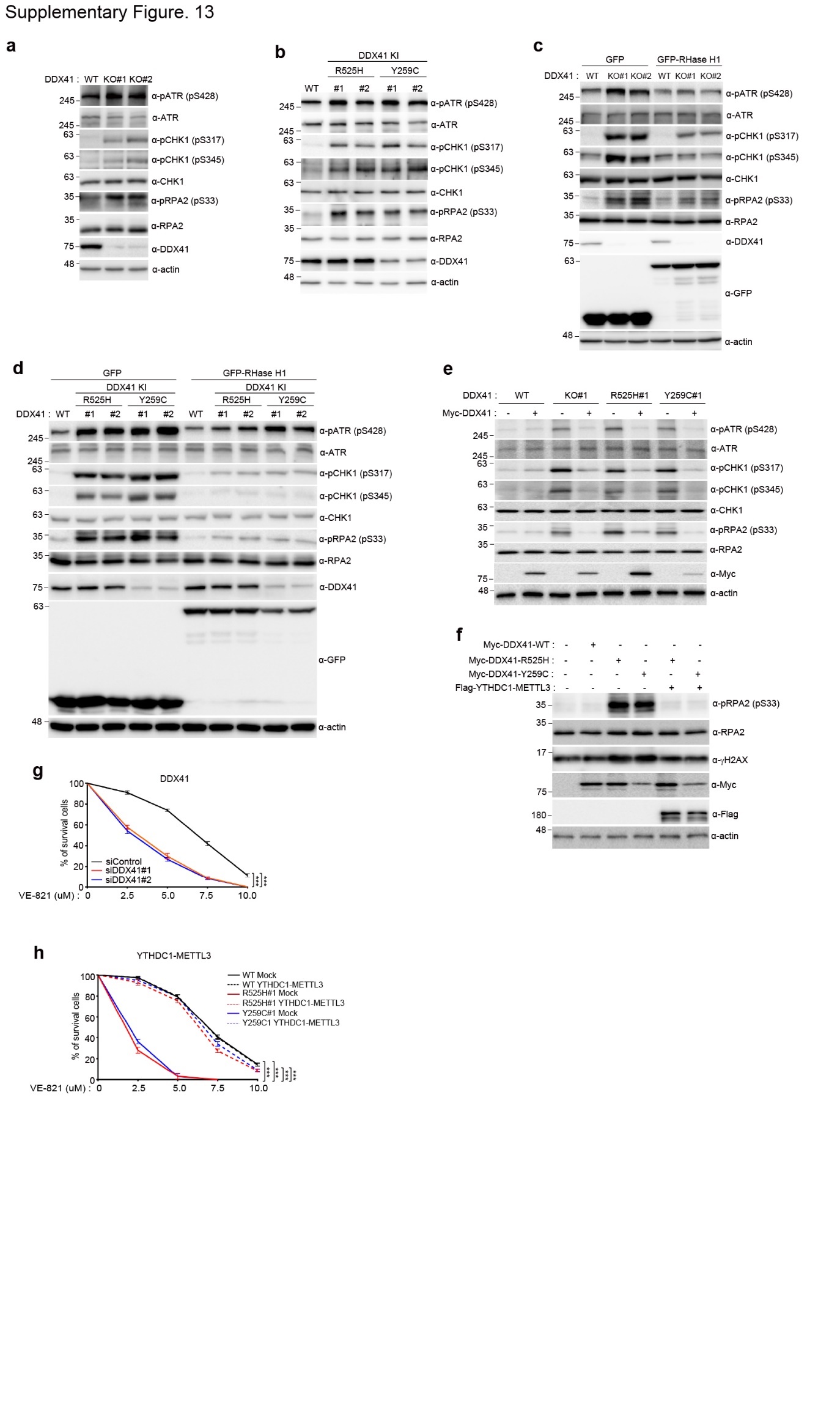
**

**Supplementary Figure S13. DDX41 regulates the ATR-CHK1 pathway.**

**(a and b)** *DDX41*WT and KO or DDX41 KI K562 cell lysates were immunoblotted with indicated antibodies. **(c and d)** *DDX41* WT and KO or KI K562 cells were transfected with GFP or GFP-RNase H1 expression plasmid. After 48 hr, cell lysates were immunoblotted with indicated antibodies. **(e)** *DDX41* WT and KO or KI K562 cells were transfected with Myc or Myc-DDX41 expression plasmid. After 72 hr, cell lysates were immunoblotted with indicated antibodies. **(f)** SKM-1 cells were transfected with the indicated expression plasmids. After 72 hr, cell lysates were immunoblotted with indicated antibodies. **(g)** Viability of control or two different DDX41 siRNA transfected SKM-1 cells following treatment with an ATR inhibitor (VE-821). 5000 cells were plated and treated with increasing concentrations of VE-821 (0, 1.25, 2.5, 5, and 10 μM). The number of cells was counted culture with VE-821 for four days. Results represent the average of three independent experiments. Error bars indicate standard deviation. Ordinary two-way ANOVA were used to determine the statistical significance (^***^*P* < 0.001). **(h)** Viability of the indicated expression plasmids transfected SKM-1 cells following treatment with an ATR inhibitor (VE-821). 5000 cells were plated and treated with increasing concentrations of VE-821 (0, 1.25, 2.5, 5, and 10 μM). The number of cells was counted culture with VE-821 for four days. Results represent the average of three independent experiments. Error bars indicate standard deviation. *P*-value was calculated based on two-way ANOVA in **(a-e)** (^***^*P* < 0.001).

Supplementary Figure S14

**
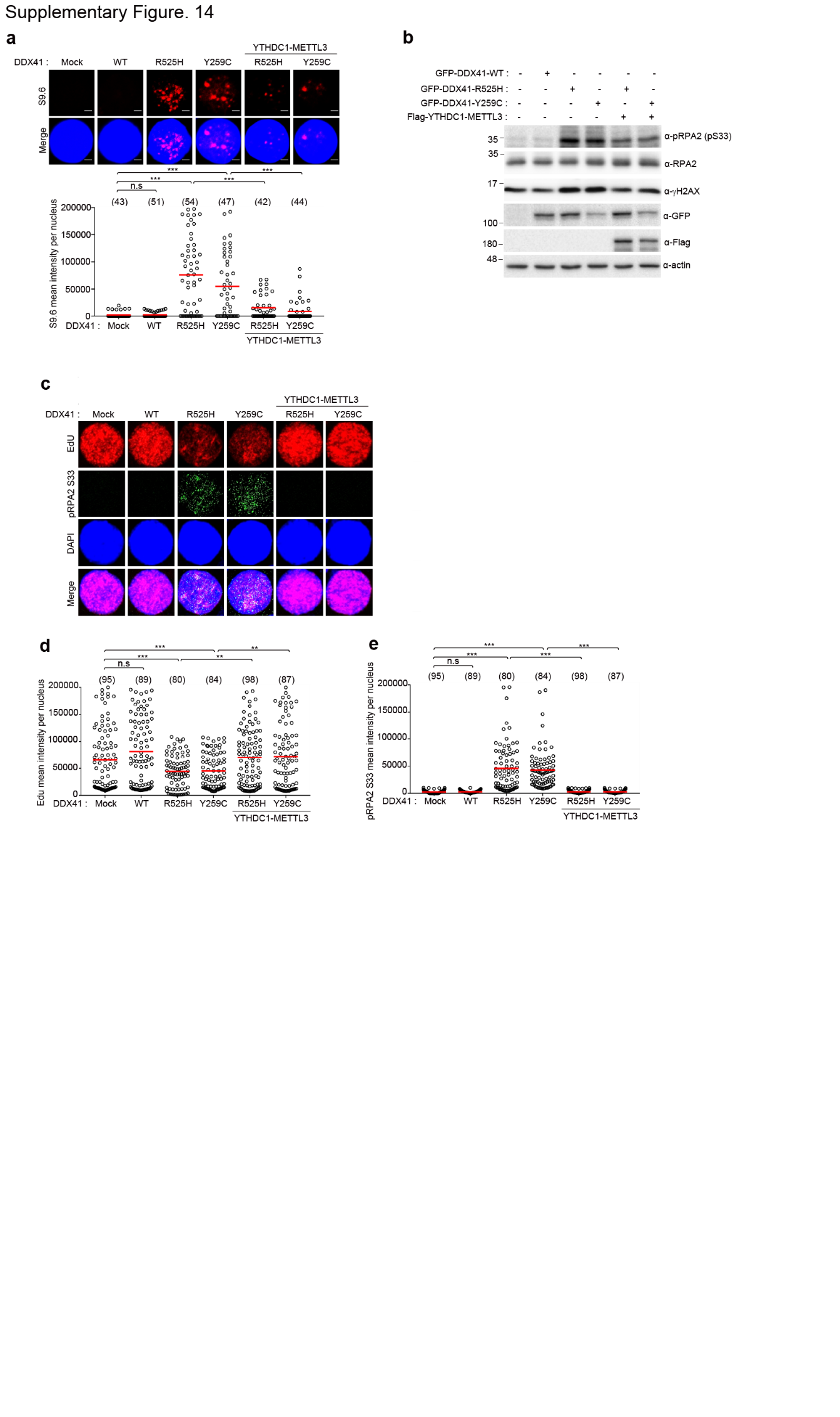
**

**Supplementary Figure S14. DDX41 regulates the S9.6 signal and RPA2 phosphorylation in *DDX41* dysfunctional primary CD34^+^ cells.**

**(a)** Human primary CD34^+^ cells were transfected with indicated expression plasmids. After 72 hr, S9.6 fluorescence intensities were determined by immunofluorescence**.** The numbers above each sample indicate the n value, which is the number of nuclei analyzed. Scale bar, 1 μm. **(b)** Human primary CD34^+^ cells were transfected with the indicated expression plasmids. After 72 hr, cell lysates were immunoblotted with indicated antibodies. **(c-e)** Human primary CD34^+^ cells were transfected with indicated expression plasmids. After 72 hr, EdU and pRPA2S33 fluorescence intensities were determined by immunofluorescence**.** The numbers above each sample indicate the n value, which is the number of nuclei analyzed. *P*-value was calculated based on one-way ANOVA in **(a, d, e)** (^**^*P* < 0.01, ^***^*P* < 0.001, n.s., non-significant). Scale bar, 1 μm.

Supplementary Figure S15

**
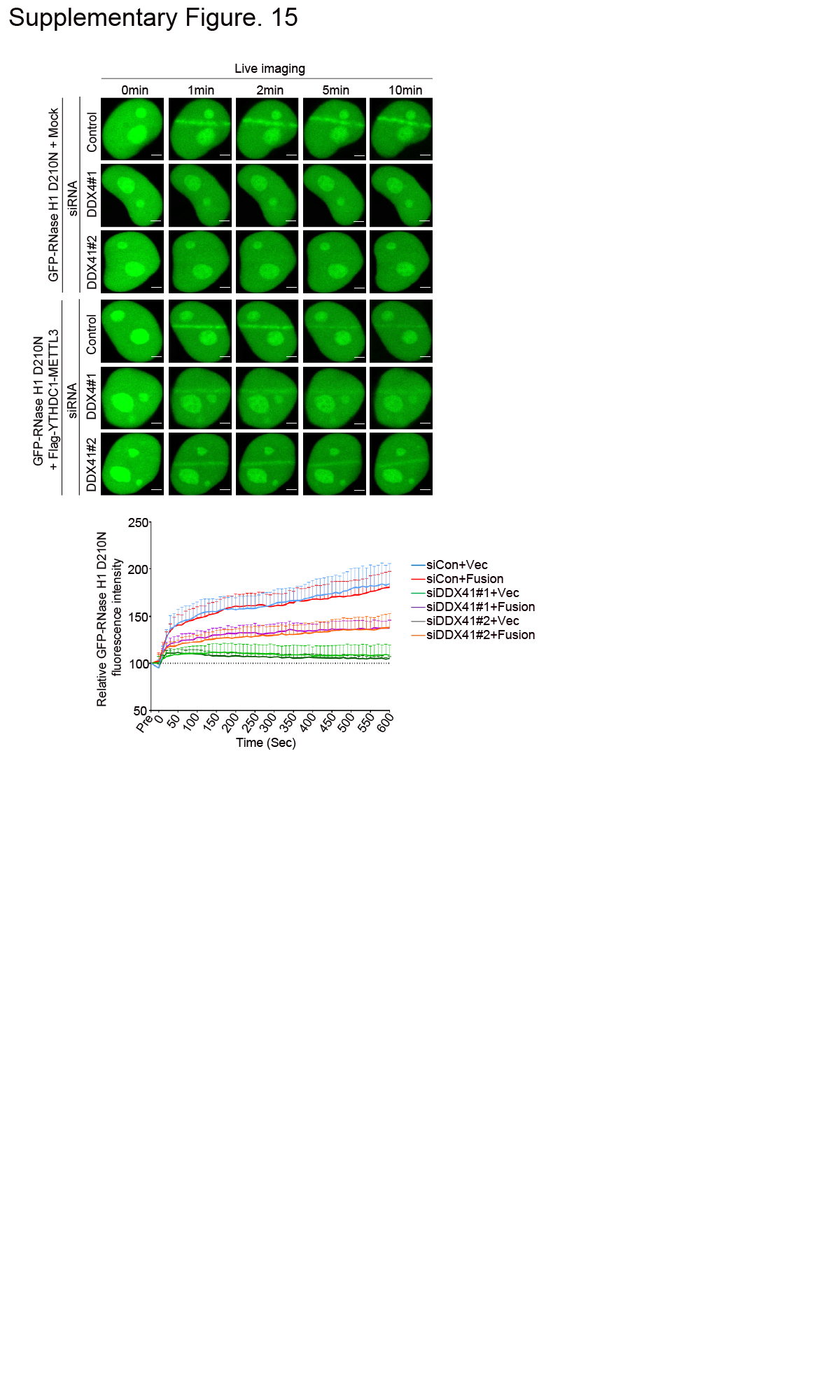
**

**Supplementary Figure S15. DDX41 promotes RNase H1 recruitment to DNA damage sites.**

Co-transfection of GFP-RNase H1 D210N with each of mock, or Flag-YTHDC1-METTL3 expression plasmid in siControl or siDDX41 transfected HeLa cells. After 24 hrs, the transfected cells were microirradiated and recruitment of GFP-RNase H1 D210N to laser strips was examined by live cell imaging. The initial intensity of region of interest (ROI) before bleaching was calculated as 100% in each cell, and then the average intensity of the laser stripes was plotted (lower panel). For this experiment, ten cells were analyzed. Data represent the mean ±SD from this experiment. Scale bar, 5 µm.

**Supplementary Table S1. Baseline characteristics of MDS patients according to presence of DDX41 mutation**

|  | *DDX41*mutation (-)* | *DDX41*mutation (+)* | *P* |
| --- | --- | --- | --- |
| Number of cases | 293 | 39 |  |
| Age, year, median (range) | 58 (19-83) | 63 (42-79) | 0.001 |
| Male sex, number (%) | 184 (62.8) | 32 (82.1) | 0.018 |
| Diagnosis, number (%)  SLD/MLD/MDS-U/del (5q)  EB1/EB2  MDS/MPN | 182 (62.1)  85 (29.0)  26 (8.9) | 20 (51.3)  19 (48.7)  0 (0.0) | 0.045 |
| Chromosome, number (%)  Normal karyotype  Abnormal karyotype | 137 (46.8)  156 (53.2) | 30 (76.9)  9 (23.1) | <0.001 |
| WBC (10^6^/L), median (range) | 3090 (640-126120) | 1760 (800-7660) | <0.001 |
| Hb (g/dL), median (range) | 8.8 (2.8-16.8) | 9.6 (4.4-13.2) | 0.525 |
| ANC (10^6^/L), median (range) | 1060 (0-78190) | 460 (80-6430) | <0.001 |
| PLT (10^9^/L), median (range) | 88 (5-654) | 84 (7-260) | 0.233 |
| BM blast, %, median (range) | 3.0 (0.0-18.0) | 4.2 (0.7-18.0) | 0.006 |
| IPSS-R  Score, median (range)  Risk by core, number (%)  < 4  ≥ 4 | 3.5 (0-10)  148 (51.0)  142 (49.0) | 4 (1-8.5)  18 (46.2)  21 (53.8) | 0.835  0.567 |
| Co-mutated genes, number (range) | 1 (0-8) | 1 (0-6) | 0.948 |
| HMT, number (%)  No  Yes | 199 (67.9)  94 (32.1) | 23 (59.0)  16 (41.0) | 0.265 |
| Response to HMT, number (%)  No  Yes | 53/92 (57.6)  39/92 (42.4) | 4/15 (26.7)  11/15 (73.3) | 0.026 |
| SCT, number (%)  No  Yes | 156 (53.2)  137 (46.8) | 23 (59.0)  16 (41.0) | 0.500 |
| Median survival, months | 108.3 | 74.6 | 0.892 |

**Abbreviations;** SLD, single lineage dysplasia; MLD, multi-lineage dysplasia; MDS-U, myelodysplastic syndrome, unclassifiable; del(5q), deletion 5q; EB, excess blasts; MDS/MPN, Myelodysplastic syndrome/myeloproliferative neoplasm; WBC, white blood cell; Hb, hemoglobin; ANC, absolute neutrophil count; PLT, platelet; BM, bone marrow; IPSS-R, revised international prognostic scoring system; HMT, hypomethylating treatment; SCT, stem cell transplantation.*Cases with *DDX41* variant of unknown significances (n=4) were not included in either group.

**Supplementary Table S2. Clinical and genomic data of MDS patients**

| Cases | Age | Sex | WHO  diagnosis | BM blast (%) | Karyotypes | *DDX41* mutation (VAF, %) | | *U2AF1* mutation (VAF, %) | Other mutation |
| --- | --- | --- | --- | --- | --- | --- | --- | --- | --- |
|  |  |  |  |  |  | Germline | Somatic |  |  |
| DDX41#1 | 63 | M | MDS-MLD | 3 | 45,X,-Y[5]/46,XY[15] | V152G (48.7) | R525H (8.8) |  | None |
| DDX41#2 | 71 | M | MDS-MLD | 2 | 46,XY[20] | V152G (50.9) | R525H (31.3) |  | None |
| DDX41#3 | 65 | F | MDS-MLD | 3 | 46,XX,del(20)(q11.2)[20]/46,XX[10] | Y259C (47.9) | R525H (8.2) |  | None |
| DDX41#4 | 63 | M | MDS-EB-2 | 15 | 46,XY[10] | c.935+4A>T (51.0) | R525H (9.5) |  | None |
| DDX41#5 | 66 | M | MDS-EB-2 | 10.9 | 46,XY[10] | V152G (49.2) |  |  | None |
| DDX41#6 | 61 | M | MDS-MLD | 3.4 | 46,XY,t(1;17)(p10;p10)c[20]^a^ | Y259C (49.2) | T377A (7.9) |  | None |
| DDX41#7 | 79 | M | MDS-EB-2 | 17 | 46,XY[20] |  | R525H (8.2) |  | None |
| DDX41#8 | 61 | M | MDS-MLD | 4.8 | 46,XY[20] | V152G (49.3) | R525H (13.6) | Q157R (16.8) | None |
| DDX41#9 | 55 | M | MDS-EB-2 | 18 | 45,XY,t(13;14)(q34;q13),-17,-20,+mar[cp7]/46,XY[13] |  | G530D (9.3) |  | *TP53, DNMT3A* |
| DDX41#10 | 61 | M | MDS-MLD | 2 | 46,XY[20] | Y259C (50.8) | A376V (11.6) |  | *DNMT3A* |
| DDX41#11 | 74 | M | MDS-U | 4 | 46,XY[20] | Y259C (48.3) |  |  | None |
| DDX41#12 | 42 | F | MDS-MLD | 4.2 | 46,XX,del(5)(q15q31)[7]/46,XX[13] | Y259C (48.4) | T227M (15.4) |  | None |
| DDX41#13 | 51 | M | MDS-EB-1 | 4 | 46,XY[20] | c.299-3C>T (49.0) |  |  | None |
| DDX41#14 | 69 | M | MDS-EB-1 | 6.4 | 46,XY[10] | Y259C (51.7) | T227M (7.8) |  | *DNMT3A* |
| DDX41#15 | 69 | M | MDS-MLD | 2 | 46,XY[20] | Y259C (50.9) | T227M (7.6) |  | *RAD50* |
| DDX41#16 | 75 | M | MDS-SLD | 1 | 46,XY[20] | R293H (49.7) |  |  | None |
| DDX41#17 | 69 | F | MDS-EB-1 | 8 | 46,XX[5] | Y259C (48.5) |  |  | *ATM, EZH2* |
| DDX41#18 | 70 | M | MDS-MLD | 1 | 47,XY,+8[2]/47,idem,del(11)(q13q23)[3]/46,XY[15] | V152G (48.2) | R525H (29.8) |  | *ASXL1* |
| DDX41#19 | 60 | M | MDS-EB-1 | 6.7 | 46,XY[20] | Y259C (45.5) | R525H (9.4) |  | None |
| DDX41#20 | 53 | M | MDS-EB-2 | 15 | 46,XY,del(20)(q13.1)[3]/46,XY[17] | c.935+4A>T (48.2) |  |  | None |
| DDX41#21 | 51 | M | MDS-MLD | 2 | 46,XY[20] | V152G (50.0) | K494E (31.6) |  | None |
| DDX41#22 | 75 | F | MDS-MLD | 3 | 46,XX[20] | Y259C (51.7) |  |  | None |
| DDX41#23 | 71 | M | MDS-EB-2 | 15 | 46,XY[20] | A500Cfs (47.4) | G530D (8.7) |  | *ASXL1* |
| DDX41#24 | 72 | M | MDS-EB-2 | 15 | 46,XY[20] | Y259C (49.9) | R525H (6.0) |  | *STAG2* |
| DDX41#25 | 71 | F | MDS-EB-1 | 7 | 46,XX[20] | V152G (50.3) | P379L (26.1) |  | None |
| DDX41#26 | 66 | F | MDS-RS-MLD | 1 | 46,XX[20] | V152G (49.5) | R525H (30.3) |  | *GNB1* |
| DDX41#27 | 51 | F | MDS-EB-1 | 6 | 46,XX[20] | M509I (49.0) |  |  | *SETBP1, STAG2, ASXL1, DNMT3A, KDM6A* |
| DDX41#28 | 44 | M | MDS-MLD | 1 | 46,XY | T214Pfs (45.6) | P321L (30.8) |  | *SRSF2* |
| DDX41#29 | 62 | M | MDS-EB-1 | 9 | 46,XY,inc[4] | Y259C (51.5) | R525H (2.1) |  | *SRSF2* |
| DDX41#30 | 54 | M | MDS-MLD | 2 | 46,XY[20] | Y259C (52.9) | P321L (14.7) |  | None |
| DDX41#31 | 66 | M | MDS-MLD | 0.7 | 46,XY[20] | V152G (47.5) | G530D (27.1) |  | *ZRSR2* |
| DDX41#32 | 58 | M | MDS-EB-1 | 9 | 46,XY[20] | V152G (44.9) | E345D (15.2) |  | None |
| DDX41#33 | 60 | M | MDS-SLD | 4 | 46,XY[20] | S217Ifs (50.0) | L390H (35.2) |  | None |
| DDX41#34 | 64 | M | MDS-MLD | 1.1 | 47,XY,+8[7]/46,XY[13] | Y259C (49.7) |  |  | None |
| DDX41#35 | 61 | M | MDS-EB-1 | 7 | 47,XY,+8[6]/46,XY[14] | A500Cfs (47.0) | R525H (5.5) |  | *PHF6* |
| DDX41#36 | 66 | M | MDS-EB-2 | 17 | 46,XY[20] | A500Cfs (48.5) | G530D (4.0) |  | None |
| DDX41#37 | 69 | M | MDS-EB-1 | 7 | 46,XY[20] | c.935+4A>T (52.9) | G530S (5.2) |  | *ZRSR2* |
| DDX41#38 | 55 | M | MDS-EB-1 | 5 | 46,XY[1] | c.935+4A>T (48.3) |  |  | None |
| DDX41#39 | 60 | M | MDS-SLD | 1 | 46,XY,inv(9)(p12q13)[20]^a^ | Y259C (50.2) | T227M (5.7) |  | None |
| U2AF1#1 | 41 | M | MDS-EB-1 | 7 | 46,XY[20] | None | None | S34F (42.6) | *ASXL1, EZH2* |
| U2AF1#2 | 73 | M | MDS-EB-1 | 4 | 47,XY,+8[20] | None | None | S34F (42.6) | *DNMT3A, TERT, BCOR, STAG2* |
| U2AF1#3 | 50 | M | MDS-EB-1 | 5 | 47,XY,+8[19]/46,XY[1] | None | None | S34Y (44.3) | *ASXL1, SF3A1, RUNX1, TET2* |
| U2AF1#4 | 61 | M | MDS-EB-1 | 5 | 47,XY,+8[20] | None | None | S34F (42.6) | *CBL, IRF1* |
| Non-DDX41/ U2AF1#1 | 63 | F | MDS-EB-1 | 8 | 46,XX,del(7)(q22q32)[18]/46,XX[2] | None | None | None | None |
| Non-DDX41/  U2AF1#2 | 54 | M | MDS-EB-1 | 9 | 46,XY,t(?17;22)(p13;q13)[10]/46,XY,del(20)(q11.2)[3]/46,idem,del(20)[3]/46,XY[4] | None | None | None | None |
| Non-DDX41/  U2AF1#3 | 69 | F | MDS-EB-1 | 9 | 46,XX[20] | None | None | None | None |
| Non-DDX41/  U2AF1#4 | 67 | M | MDS-EB-1 | 6.4 | 46,XY[20] | None | None | None | None |
| Non-DDX41/  U2AF1#5 | 49 | M | MDS-EB-1 | 6 | 46,XY,t(1;20)(p36.1;q13.3)[20] | None | None | None | *BCOR* |
| Non-DDX41/  U2AF1#6 | 59 | M | MDS-EB-1 | 9 | 44,XY,-5,del(7)(q22q35),add(12)(q24.2),-16,der(19)t(1;19)(p22;p13.3)[8]/46,XY[12] | None | None | None | *TP53* |
| Non-DDX41/  U2AF1#7 | 60 | M | MDS-EB-1 | 9 | 46,XY[20] | None | None | None | *IDH2, BCOR, DNMT3A, ASXL1* |

**Abbreviations;** WBC, white blood cell; ANC, absolute neutrophil count; Hb, hemoglobin; PLT, platelet; BM, bone marrow; HMA, hypomethylating agent; SLD, single lineage dysplasia; MLD, multi-lineage dysplasia; MDS-U, myelodysplastic syndrome, unclassifiable; del(5q), deletion 5q; EB, excess blasts; MDS/MPN, Myelodysplastic syndrome/myeloproliferative neoplasm; IPSS-R, revised international prognostic scoring system; VAF, variant allele frequency

^a^ Confirmed constitutional abnormality was categorized as normal karyotype.

**Supplementary Table S3: The oligonucleotides used in this study.**

| **Name** | **Sequence** |
| --- | --- |
| DDX41 siRNA #1 | 5’-CUAAGAGUGCCCUUGUAAAUU-3’ |
| DDX41 siRNA #2 | 5’-CCAUCUUCUCCUACUUCAAUU-3’ |
| Control siRNA | 5’-UUCAAUAAAUUCUUGAGGUUU-3’ |
| DDX41 R525H ssDNA | 5’-CTGTCCTTCTCTCTGCAGTACACCGGATTGGCCGCACCGGTCACTCGGGAAACACAGGCATCGCCACTACCTTCATCAACAAA-3’ |
| DDX41_R525H gRNA | 5’-GAGCGCCCGGTGCGGCCAAT-3’ |
| DDX41 Y259C ssDNA | 5’-AACAAGAGAAGAGGTTACCCTTCTCAAAGCGCGAGGGGCCaTgcGGACTCATCATCTGCCCCTCGGTAAGATAGGCTGGCCTGGA-3’ |
| DDX41_Y259C gRNA | 5’-GGGGCAGATGATGAGTCCATAGG-3’ |
| hDDX41 KO#10 gRNA#1 F | 5'-CACCGATGTGCCGTTACGGCAGCGC-3' |
| hDDX41 KO#10 gRNA#1 R | 5'-AAACGCGCTGCCGTAACGGCACATC-3' |
| hDDX41 KO#10 gRNA#2 F | 5’-CACCGCTGCTGCAGCGAAGACGCAA-3’ |
| hDDX41 KO#10 gRNA#2 R | 5'-AAACTTGCGTCTTCGCTGCAGCAGC-3’ |
| hDDX41 KO#32 gRNA#1 F | 5'-CACCGTTACCCTTCTCAAAGCGCG-3' |
| hDDX41 KO#32 gRNA#1 R | 5'-AAACCGCGCTTTGAGAAGGGTAAC-3' |
| hDDX41 KO#32 gRNA#2 F | 5'-CACCGGCCCCTCGCGCTTTGAGAA-3' |
| hDDX41 KO#32 gRNA#2 R | 5'-AAACTTCTCAAAGCGCGAGGGGCC-3' |
| hDDX41 KO sequencing F | 5’-ATATGAATTCAACGGAAGGTAGGAGAGAG-3’ |
| hDDX41 KO sequencing R | 5’-ACACCTCGAGATGTCAGACAGATACCAAAACG-3’ |
| hDDX41 Myc, SFB, GFB, mCherry N1 vector F | 5’-GCGCGCGAATTCATGGAGGAGTCGGAACCCGAA-3’ |
| hDDX41 Myc, SFB vector R | 5’-GCGCGCGGATCCTCAGAAGTCCATGGAGCTGTG-3’ |
| hDDX41 GFP, mCherry N1 vector R | 5’-GCGCGCGGATCCGCGAAGTCCATGGAGCTGTGGGC-3’ |
| hDDX41 D1 GFP F | 5’-AGCCGCTCCGAGGCGGAAATGAAGTTTCCTGCAGCC-3’ |
| hDDX41 D1 GFP R | 5’-GGCTGCAGGAAACTTCATTTCCGCCTCGGAGCGGCT-3’ |
| hDDX41 D2 GFP F | 5’-ATCAAGAGCTTCAAGGAAATCCAGGAGGTAGAATAT-3’ |
| hDDX41 D2 GFP R | 5’-ATATTCTACCTCCTGGATTTCCTTGAAGCTCTTGAT-3’ |
| hDDX41 D3 GFP F | 5’-GCTGCCAGCCTGGATGTCACCTTCATCAACAAAGCG-3’ |
| hDDX41 D3 GFP R | 5’-CGCTTTGTTGATGAAGGTGACATCCAGGCTGGCAGC-3’ |
| hDDX41 D4 GFP F | 5’-GCGCGCGAATTCATGGAGGAGTCGGAACCCGAA-3’ |
| hDDX41 D4 GFP R | 5’-GCGCGCGGATCCGCAGTGGCGATGCCTGTGTT-3’ |
| hDDX41 N term (1-408 a.a) Myc vector F | 5’-GCGCGCGAATTCATGGAGGAGTCGGAACCCGAA-3’ |
| hDDX41 N term (1-408 a.a) Myc vector R | 5’-GCGCGC GGATCCTCAGACATCCAGGCTGGCAGC-3’ |
| hDDX41 C term (409-622 a.a) Myc vector F | 5’-GCGCGCGAATTCATGCCCGAACGGAAGCGGGCTCGCACCGACATCCAGGAGGTAGAATAT-3’ |
| hDDX41 C term (409-622 a.a) Myc vector R | 5’-GCGCGCGGATCCTCAGAAGTCCATGGAGCTGTG-3’ |
| hDDX41 A1 Myc vector F | 5’-GCGCGCGAATTCATGGAGGAGTCGGAACCCGAA-3’ |
| hDDX41 A1 Myc vector R | 5’-GCGCGCGGATCCTCATTCCTTGAAGCTCTTGAT-3’ |
| hDDX41 A2 (DEAD domain) Myc, GFP vector F | 5’-GCGCGCGAATTCATGCCCGAACGGAAGCGGGCTCGCACCGACATGAAGTTTCCTGCAGCC-3’ |
| hDDX41 A2 (DEAD domain) Myc vector R | 5’-GCGCGCGGATCCTCAGACATCCAGGCTGGCAGC-3’ |
| hDDX41 DEAD domain GFP vector R | 5’-GCGCGCGGATCCGCGACATCCAGGCTGGCAGC-3’ |
| hDDX41 A3 Myc vector F | 5’-GCGCGCGAATTCATGCCCGAACGGAAGCGGGCTCGCACCGACATCCAGGAGGTAGAATAT-3’ |
| hDDX41 A3 Myc vector R | 5’-GCGCGCGGATCCTCAAGTGGCGATGCCTGTGTT-3’ |
| hDDX41 A4 Myc vector F | 5’-GCGCGCGAATTCATGCCCGAACGGAAGCGGGCTCGCACCGACACCTTCATCAACAAAGCG-3’ |
| hDDX41 A4 Myc vector R | 5’-GCGCGCGGATCCTCAGAAGTCCATGGAGCTGTG-3’ |
| hDDX41 Y259C (c.766A>G) GFP vector F | 5’-TCAAAGCGCGAGGGGCCCTGTGGACTCATCATCTGCCCC-3’ |
| hDDX41 Y259C (c.766A>G) GFP vector R | 5’-GGGGCAGATGATGAGTCCACAGGGCCCCTCGCGCTTTGA-3’ |
| hDDX41 R525H (c.1574G>A) GFP vector F | 5’-CGGATTGGCCGCACCGGGCACTCGGGAAACACAGGCATC-3’ |
| hDDX41 R525H (c.1574G>A) GFP vector R | 5’-GATGCCTGTGTTTCCCGAGTGCCCGGTGCGGCCAATCCG-3’ |
| hMettl3 SFB vector F | 5’-GCGCGCGTCGACGCATGTCGGACACGTGGAGC-3’ |
| hMettl3 SFB vector R | 5’-GCGCGCCCGCGGCTATAAATTCTTAGGTTT-3’ |
| hMettl3 D1 SFB vector F | 5’-ATGTCGGACACGTGGAGCTCTCCAAAAAAAAAAAGAAAAGTTTCCATTGTTGAAAAATTTCGC-3’ |
| hMettl3 D1 SFB vector R | 5’-GCGAAATTTTTCAACAATGGAAACTTTTCTTTTTTTTTTTGGAGAGCTCCACGTGTCCGACAT-3’ |
| hMettl3 D2 SFB vector F | 5’-ACAACAGCCAAGGAACAACACACGCCAAGCCAGGAG-3’ |
| hMettl3 D2 SFB vector R | 5’-CTCCTGGCTTGGCGTGTGTTGTTCCTTGGCTGTTGT-3’ |
| hMettl3 D3 SFB vector F | 5’-GCCCCTGGCAGCAAAGACAAACCTAAGAATTTATAG-3’ |
| hMettl3 D3 SFB vector R | 5’-CTATAAATTCTTAGGTTTGTCTTTGCTGCCAGGGGC-3’ |
| hMettl3 D4 SFB vector F | 5’-GCGCGCGAATTCATGCCAAAAAAAAAAAGAAAAGTTCACACGCCAAGCCAG GAG-3’ |
| hMettl3 D4 SFB vector R | 5’-GCGCGCGTCGACCTATAAATTCTTAGGTTT-3’ |
| hMettl14 SFB vector F | 5’-GCGCGCGAATTCATGGATAGCCGCTTGCAG-3’ |
| hMettl14 SFB vector R | 5’-GCGCGCGGATCCTTATCGAGGTGGAAAGCC-3’ |
| hMettl14 D1 SFB vector F | 5’-GCGCGCGAATTCATGCCAAAAAAAAAAAGAAAAGTTACACAGAGCTTAAATCCC-3’ |
| hMettl14 D1 SFB vector R | 5’-GCGCGCGGATCCTTATCGAGGTGGAAAGCC-3’ |
| hMettl14 D2 SFB vector F | 5’-AGTACTTTTCTTAAGGGAAAATCTGACCGAGGAGGT-3’ |
| hMettl14 D2 SFB vector R | 5’-ACCTCCTCGGTCAGATTTTCCCTTAAGAAAAGTACT-3’ |
| hMettl14 D3 SFB vector F | 5’-GCGCGCGAATTCATGGATAGCCGCTTGCAG-3’ |
| hMettl14 D3 SFB vector R | 5’-GCGCGCGGATCCTTAAGATTTGGGAGGAGGCGA-3’ |
| hMettl14 D4 SFB vector F | 5’-GCGCGCGAATTCATGCCAAAAAAAAAAAGAAAAGTTACACAGAGCTTAAATCCC-3’ |
| hMettl14 D4 SFB vector R | 5’-GCGCGCGGATCCTTAAGATTTGGGAGGAGGCGA-3’ |
| hYTHDC1 SFB, Myc, GFP vector F | 5’-GCGCGCGAATTCATGGCGGCTGACAGTCGGG-3’ |
| hYTHDC1 SFB vector R | 5’-GCGCGCCCGCGGTTATCTTCTATATCGACC-3’ |
| hYTHDC1 Myc vector R | 5’-GCGCGCGGTACCTTATCTTCTATATCGACC-3’ |
| hYTHDC1 GFP vector R | 5’-GCGCGCGGTACCCCTCTTCTATATCGACCTCTCTCCCC-3’ |
| hYTHDC1 D1 SFB vector F | 5’-ATGGCGGCTGACAGTCGG GATGAGCAAGGGAACAAC-3’ |
| hYTHDC1 D1 SFB vector R | 5’-GTTGTTCCCTTGCTCATCCCGACTGTCAGCCGCCAT-3’ |
| hYTHDC1 D2 SFB vector F | 5’-GGCAGCAGTGGTTCTTCTTCTGAATCTGTTTCCTTC-3’ |
| hYTHDC1 D2 SFB vector R | 5’-GAAGGAAACAGATTCAGAAGAAGAACCACTGCTGCC-3’ |
| hYTHDC1 D3 SFB vector F | 5’-GAGGCCAGTGACTCTGGTACCAGTAAACTCAAATAT-3’ |
| hYTHDC1 D3 SFB vector R | 5’-ATATTTGAGTTTACTGGTACCAGAGTCACTGGCCTC-3’ |
| hYTHDC1 D4 SFB vector F | 5’-GCTGTCCGAAAAGATCAAAAAATGCGTCACAAGAGA-3’ |
| hYTHDC1 D4 SFB vector R | 5’-TCTCTTGTGACGCATTTTTTGATCTTTTCGGACAGC-3’ |
| hYTHDC1 D5 SFB vector F | 5’-TTGTATCAGGTCATTCATAATGATTATGTGAGGGAA-3’ |
| hYTHDC1 D5 SFB vector R | 5’-TTCCCTCACATAATCATTATGAATGACCTGATACAA-3’ |
| hYTHDC1 D6 SFB vector F | 5’-TTTTTAAATGGGTCCTACCATCCAGTACCACATGAA-3’ |
| hYTHDC1 D6 SFB vector R | 5’-TTCATGTGGTACTGGATGGTAGGACCCATTTAAAAA-3’ |
| hYTHDC1 D7 SFB vector F | 5’-CCCCCTTACTCAGGACATGGTCGATATAGAAGATAA-3’ |
| hYTHDC1 D7 SFB vector R | 5’-TTATCTTCTATATCGACCATGTCCTGAGTAAGGGGG-3’ |
| hYTHDC1 A4 Myc vector F | 5’-GCGCGCGAATTCATGCCAAAAAAAAAAAGAAAAGTTACCAGTAAACTCAAATAT-3’ |
| hYTHDC1 A4 Myc vector R | 5’-GCGCGCGGTACCTTAATGAATGACCTGATACAA-3’ |
| hYTHDC1-DDX41 flag vector F | 5’-GCGCGCACCGGTGCCACCATGGACTACAAGGATGACGACGACAAG GATTACAAAGATGACGACGATAAGGACTACAAGGATGACGACGACAAG ATGGCGGCTGACAGTCGGGAG-3’ |
| hYTHDC1-DDX41 flag vector R | 5’-GCGCGCGAATTCCGATCCGCCACCGCCAGAGCCACCTCCGCCTGAACCGCCTCCACCTCTTCTATATCGACCTCTCTC-3’ |
| hYTHDC1-METTL3 flag vector F | 5’-GCGCGCACCGGTGCCACCATGGACTACAAGGATGACGACGACAAGGATTACAAAGATGACGACGATAAGGACTACAAGGATGACGACGACAAG ATGGCGGCTGACAGTCGGGAG-3’ |
| hYTHDC1-METTL3 flag vector R | 5’-GCGCGCAAGCTTGCCGATCCGCCACCGCCAGAGCCACCTCCGCCTGAACCGCCTCCACCTCTTCTATATCGACCTCTCTC-3’ |

**Supplementary Table S4. List of synthesized oligomers**

| **Name** | **Sequence** |
| --- | --- |
| R-loop oligo1* | 5’-[Cy5]-GCC AGG GAC GAG GTG AAC CTG CAG GTG GGC **GGC TAC TAC TTA GAT GTC ATC CGA GGC TTA T**TG GTA GAA TTC GGC AGC GTC ATG C GA CGG C-3’ |
| R-loop oligo2* | 5’-GCC GTC GCA TGA CGC TGC CGA ATT CTA CCA **CGC GAT TCA TAC CTG TCG TGC CAG CTG CTT T**GC CCA CCT GCA GGT TCA CCT CGT CCC TGG C-3’ |
| R-loop RNA | 5’-[Cy3]-GCA GCU GGC ACG ACA GGU AUG AAU C-3’ |
| m6A RNA** | 5’-[Cy3]-GC***A*** GCU GGC ***A***CG ACA GGU AUG A***A***U C-3’ |
| D-loop DNA | 5’-GCA GCT GGC ACG ACA GGT ATG AAT C-3’ |
| Homoduplex | 5’-[Cy5]-GCC AGG GAC GAG GTG AAC CTG CAG GTG GGC AAA GCA GCT GGC ACG ACA GGT ATG AAT CGC GTG GTA GAA TTC GGC AGC GTC ATG CGA CGG C-3’ |
| Hybrid DNA | 5’-CCC ATA CCG TAT AAC CAT TTG GCT GTC CAA GCT CCG GGT-3’ |
| Hybrid RNA | 5’-[Cy5]-ACC CGG AGC UUG GAC AGC CAA AUG GUU AUA CGG UAU GGG-3’ |
| dT_30_ | 5’-TTT TTT TTT TTT TTT TTT TTT TTT TTT TTT-3’ |
| PTJ 1 | 5’-[Cy5]GCCTCGCTGCCGTCGCCA[Biotin]-3’ |
| PTJ 2 | 5’-TGGCGACGGCAGCGAGGCTTTTTTTTTTTTTTTTTT[Cy3]-3’ |

* Bold represents bubble for R-loop.

** Bold italic A represents N6 methylated A.

**Supplementary Table S5. Annealing for diverse DNA or RNA substrates**

|  | Components and concentration | Buffer |
| --- | --- | --- |
| dsDNA | R-loop oligo 1: 6 uM  Homoduplex: 5 uM | 25 mM Tris-HCl [8.0]  100 mM NaCl |
| R-loop | R-loop oligo 1: 6 uM  R-loop oligo 2: 5 uM  R-loop RNA: 5 uM |  |
| R-loop(m6A) | R-loop oligo 1: 6 uM  R-loop oligo 2: 5 uM  m6A RNA: 5 uM |  |
| D-loop | R-loop oligo 1: 6 uM  R-loop oligo 2: 5 uM  D-loop DNA: 5 uM |  |
| Bubble | R-loop oligo 1: 6 uM  R-loop oligo 2: 5 uM |  |
| Hybrid | Hybrid DNA: 5 uM  Hybrid RNA: 5 uM |  |
| ssDNA | dT_30_: 5 uM |  |
| Primer-template junction | PTJ 1: 6 uM  PTJ 2: 5 uM |  |
